# Analysing concordance between MUAC, MUACZ, and WHZ in diagnosing acute malnutrition among children under five in Somalia

**DOI:** 10.7189/jogh.15.04258

**Published:** 2025-12-12

**Authors:** Sydney Garretson, Shelley Walton, Kemish Kenneth Alier, Samantha Grounds, Qundeel Khattak, Said Aden Mohamoud, Abdullahi Abdulle Farah, Farhan Mohamed Mohamud, Abdullahi Muse Mohamoud, Adan Mahdi, Meftuh Omer Ismail, Mohamed Billow Mahat, Fabrizio Loddo, Marina Tripaldi, Nadia Akseer

**Affiliations:** 1 Johns Hopkins University, Department of International Health, Baltimore, Maryland, USA; 2 Save the Children International, London, UK; 3 Save the Children Somalia, Mogadishu, Somalia; 4 Federal Ministry of Health Somalia, Nutrition Section, Mogadishu, Somalia

## Abstract

**Background:**

Acute malnutrition, also known as wasting, affected an estimated 45 million children under five (CU5) globally in 2023. Wasting is measured using a child’s mid upper arm circumference (MUAC), weight-for-height z-score (WHZ), or nutritional oedema. In low-resource contexts, MUAC is often the only measurement used to regularly screen for malnutrition, but recent research suggests MUAC alone fails to diagnose 25–80% of WHZ-wasted children. Mid upper arm circumference, an age-adjusted MUAC z-score, may identify additional cases missed by MUAC alone.

**Methods:**

This was a secondary analysis of 1408 CU5 enrolled in a cluster-randomised controlled trial in two high-wasting regions of Somalia. This analysis explored wasting prevalences, concordance between indicator pairs, linear regression modelling, and receiver operating characteristic (ROC) analysis using WHZ as a reference standard to examine alternative MUAC and MUACZ thresholds.

**Results:**

Wasting prevalence was 1.5% by MUAC, 8.5% by MUACZ, and 14.8% by WHZ. Mid upper arm circumference alone failed to identify 94% of WHZ-wasted children. There was slight concordance between MUAC and WHZ (kappa (κ) = 0.089) and fair concordance between MUACZ and WHZ (κ = 0.385). Linear regression indicated that MUAC, age, sex, and stunting were all statistically significant variables for estimating child WHZ. Using WHZ as a reference standard, a MUAC of 13.7cm and MUACZ of –0.9 both accurately diagnosed >82% of wasted children ages 9–23 months. A MUAC of 14.4 cm and MUACZ of −1 both accurately diagnosed >76% of wasted children ages 24–59 months.

**Conclusions:**

Poor concordance between MUAC and WHZ should prompt review of wasting measurement guidelines for this population. Stratified analyses and regression modelling showed increased MUAC with age among CU5. MUACZ or age-specific MUAC thresholds may identify more truly wasted children, improving coverage of treatment interventions. Policymakers should examine how adapting screening guidelines impacts health facilities and treatment availability. Future studies should consider long-term outcomes and mortality associated with increased thresholds of MUAC and MUACZ.

**Registration:**

The cluster-RCT is registered at ClinicalTrials.gov, ID: NCT06642012.

A recent UNICEF report includes Somalia among the 15 countries worldwide with the highest rates of malnutrition, food insecurity, and food poverty [1]. The Integrated Food Security Phase Classification (IPC) estimates that in 2024, 1.7 million children under five (CU5) in Somalia will be acutely malnourished, 430 000 of whom will be severely wasted [2].

The World Health Organization (WHO) has established guidelines for diagnosing acute malnutrition based on the presence of oedema and two anthropometric criteria, mid-upper arm circumference (MUAC) and weight-for-height z-score (WHZ) [3]. Weight-for-height z-scores compare a child’s weight to another child of the same height from a standard reference population and is scored as the number of standard deviations (SD) from the median reference WHZ [4]. Definitions for malnutrition diagnosis across indicators, including stratification into moderate and severe acute malnutrition (MAM and SAM, respectively), may be found in Table S1, Panel A in the **Online Supplementary Document****.**

MUAC was initially recommended in the 1960s for assessing malnutrition in CU5 due to its strong correlation with muscle mass [5–7]. Over time, research revealed age-related variations in MUAC, prompting a shift towards weight-for-height as a more precise measure of child nutrition status [5,8]. However, the use of MUAC tape for malnutrition diagnosis persists in many low-resource settings and community health screenings due to its low cost and ease of measurement [5,9-12]

In Somalia, community-level malnutrition screening utilises MUAC and/or oedema as criteria for health care facility treatment referrals [11]. The child’s weight and height are measured upon admission to a health centre for treatment but are not used for screening due to resource constraints, measurement complications, and limited awareness and policy enforcement from both government and non-government organisations.

Recent population-level representative surveys conducted in humanitarian settings have shown that MUAC wasting prevalences underestimate WHZ wasting prevalences by 5–10 percentage points and diagnose different children as wasted [12–15]. Leidman 2019 and Roberfroid 2015 found that among children identified as wasted by either MUAC or WHZ, only 15–30% were identified by both criteria [12,16]. Female, younger, and stunted children are more likely to be diagnosed as malnourished by MUAC [16–19]. Cohen’s kappa has been used in several studies to assess concordance between MUAC and WHZ: concordance results ranged from 0.21 to 0.35, indicating fair concordance at best [14,17,20]. Receiver-operating characteristic analysis has been used to evaluate adjusted MUAC thresholds, finding that MUAC cutoffs greater than 13.5cm have shown increased sensitivity for diagnosing WHZ-wasted CU5 [14,17,18].

Prior studies have also evaluated MUACZ, an age-adjusted MUAC z-score as a simplified alternative measurement to resolve discrepancies between MUAC and WHZ: MUACZ categorised a higher percentage of children as wasted than both MUAC and WHZ, but the difference was not substantial enough to warrant programmatic use [12,20,21]. In a study of MUACZ in Somalia, the prevalence of acute malnutrition was more similar by WHZ and MUACZ ( ~ 16%) compared to 8% by MUAC only [22].

Due to substantial regional differences in malnutrition prevalences, Somalia appears to be a global outlier, with studies finding poor concordance and an 8–10 percentage point difference in wasting prevalences by MUAC and WHZ [12,13,22,23]. However, to our knowledge, no studies have generated recent wasting prevalences in Somalia. Other concordance studies have used national survey data sets and present population-level reviews of data collected from 1986 to 2022. This analysis supplements existing evidence with anthropometric data collected for a cluster-randomised controlled trial to generate real-time wasting prevalence estimates within six months of data collection.

The aim of this study is to examine concordance between MUAC, MUACZ, and WHZ prevalence estimates of acute malnutrition among children under five in two high wasting burden regions in Somalia, Bay and Hiran. Specific objectives were to:

1) Generate acute malnutrition prevalences using each anthropometric indicator (MUAC, MUACZ, and WHZ)

2) Compare the concordance between indicators for assessing child wasting (defined as MUAC<12.5cm, MUACZ<−2, WHZ<−2) in children ages 6 − 59 months in Somalia, stratified by two regions (Bay *vs*. Hiran) and by child age (9–23 months *vs*. 24–59 months) and

3) Examine sensitivities and specificities of alternative MUAC and MUACZ thresholds using WHZ as a reference standard.

We hypothesised low concordance between MUAC and WHZ, but improved concordance between MUACZ and WHZ, and that MUAC cutoffs above 13 cm would improve diagnostic performance compared to WHZ.

## METHODS

### Study setting and design

This study was an exploratory analysis using data from a cluster-randomised controlled trial conducted in two high wasting burden regions of Somalia, Bay and Hiran. These regions are heavily impacted by conflict and climate instability, leading to high rates of food insecurity and displacement from conflict, droughts, and flooding [24–26]. Bay and Hiran were selected for the trial based on their high maternal and child malnutrition burden and representativity of other regions in Somalia.

Data for the trial were collected from May–December 2023 for the Cash Plus for Nutrition programme implemented by Save the Children in Somalia. This cluster-randomized controlled trial enrolled CU5 and their mothers in Bay and Hiran to investigate which combinations of cash assistance are most effective and cost-effective for reducing malnutrition. Inclusion criteria for trial enrollment were registration in a Bureau for Humanitarian Assistance (BHA)-funded programme, no prior wasting by MUAC within the last 12 months, and no wasting by MUAC at baseline data collection (May 2023). A detailed description of the trial design can be found in the study’s methodology paper [27].

This exploratory analysis was selected as a key research priority of government and NGO stakeholders and was a pre-specified objective for the trial. Given limited studies in humanitarian and conflict-affected settings, the anthropometric measurements taken by this trial offer more precise data for studying measurement concordance.

### Outcomes

Table S1, Panel A in the **Online Supplementary Document** above contains all child outcomes. Child MUAC was measured using standard MUAC tape, to the nearest 0.1cm. Child weight was measured to the nearest 0.01 kg, using a UNICEF electronic stand-on floor scale. Child height or length was measured to the nearest 0.1 cm, using an ICLEAR Kid Growth Wooden Height-Length Measuring Board. Study enumerators were thoroughly trained in best anthropometry practices using SMART methodology and each measurement was taken twice before recording [28]. Where possible, enumerators also completed age verification for study participants.

### Analysis approach

This is a secondary analysis of endline data (collected December 2023) emerging from the cluster-randomised trial. Data were analysed as a cohort of children.

The initial data set included 1475 children ages 9–62 months. This analysis was restricted to CU5, so children ages 60–62 months were excluded (n = 21). Using STATA’s zanthro module, the 2007 WHO Growth Standards were imported to transform anthropometric data into weight-for-height, height-for-age, and MUAC z-scores [29]. The 2007 WHO Growth Charts and zanthro command allowed us to calculate MUACZ for each child, which was not available using the 2006 WHO Growth Charts and the accompanying zscore06 module in STATA [29–31]. Extreme or biologically implausible WHZ (<−5 SD and >5 SD) or height-for age (HAZ) values (<−6 SD and >6 SD) were dropped from the data set (n = 46) [32]. The final analytical data set included 1408 children.

Wasting prevalences and 95% confidence intervals were calculated for wasting by MUAC, MUACZ, WHZ, and the 2013 WHO wasting management guidelines. Wasting prevalences were categorised as binary variables (wasted *vs*. not wasted) for each indicator, and additionally stratified into normal, MAM, and SAM. Definitions for these terms are found in Table S1, Panel A in the **Online Supplementary Document****.**

Given the approximately normal distributions of MUAC, MUACZ, and WHZ in the sample and linear relationships between these variables, Pearson correlation coefficients were produced. The data met necessary assumptions to perform linear regression with robust variance using WHZ as the outcome and MUAC, MUACZ, sex, age, and stunting as covariates. Stunting (HAZ<−2 SD) was included as a covariate of importance, since stunted children are especially vulnerable to malnutrition and may confound linear relationships between indicators [16].

Cohen’s kappa statistics were produced for pairwise combinations of MUAC, MUACZ, and WHZ to evaluate concordance in wasting diagnosis. Kappa values were interpreted as: ≤0 indicating no concordance, 0.01–0.20 as none to slight, 0.21–0.40 as fair, 0.41–0.60 as moderate, 0.61–0.80 as substantial, and 0.81–1.00 as near perfect concordance [33]. Concordance was assessed with dichotomous diagnoses of wasting and with stratification into SAM/MAM/Normal.

Single-point receiver operating characteristic (ROC) analysis was conducted using WHZ as a ‘reference standard’, examining sensitivities and specificities of different MUAC and MUACZ cutoffs for diagnosing wasting by WHZ. Since treating more children who are potentially wasted would be preferred over excluding truly wasted children, sensitivity was prioritised in this analysis. However, false positive diagnoses have considerable implications in practice, given that increased caseloads and overtreatment of non-wasted children will further strain health care facilities with limited resources. Thus, criteria for determining ‘optimal’ thresholds were maximising AUC and sensitivity while maintaining a false positive rate less than 35%, similarly to previous analyses [34].

The analysis was also stratified by child age and region to evaluate dose-response relationships since wasting prevalences varied significantly (Table S5, Panel A and Table S6, Panel A in the **Online Supplementary Document**). Age categories 9–23 months and 24–59 months were compared since this cutoff was used consistently in prior research [12,15,18]. Additionally, age was further stratified into four categories (9–23 months, 24–35 months, 36–47 months, and 48–59 months) to examine changes in child growth by year.

The two-sided alpha was set to 0.05 *a priori* and a *P*-value <0.05 indicated statistical significance. All analyses were performed using STATA version 18.0 (StataCorp, College Station, TX, USA).

## RESULTS

The following results are from the endline data set for this research study. Results were consistent from midline to endline with minimal differences in concordance and ROC analysis results. Midline results are included in the supplementary materials (Annexes S8–16 in the **Online Supplementary Document**).

### Descriptive statistics

Descriptive statistics were performed for demographic and anthropometric data (**Table 1**). There was a significantly larger proportion of female children in Bay than in Hiran. Mean child age and the proportion of children ages 24–59 months was greater in Hiran than in Bay. Mean MUAC, MUACZ, and WHZ were all lower in Hiran than Bay. Stunting prevalence was three times greater in Bay than in Hiran. There were moderate positive correlations between MUAC and WHZ (rho (ρ) = 0.55), but stronger correlations between both MUAC and MUACZ, (ρ = 0.85), and MUACZ and WHZ (ρ = 0.65) (Figure S2, Panels A–C in the **Online Supplementary Document**).

**Table 1:** Sample characteristics

	Total sample (n = 1408)	Hiran (n = 794)	Bay (n = 614)	*P*-values
**Demographic characteristics**				
Child sex, n (%)				
*Male*	699 (49.6%)	415 (52.3%)	284 (46.3%)	0.025
*Female*	709 (50.4%)	379 (47.7%)	330 (53.7%)	
Child age, x̄ (SD)	36.82 (13.79)	38.79 (13.72)	34.27 (13.47)	<0.001
*9–23 mo, n (%)*	270 (19.2%)	131 (16.5%)	139 (22.6%)	0.004
*23–59 mo, n (%)*	1138 (80.8%)	663 (83.5%)	475 (77.4%)	
Child age – 4 categories*				
*9–23 mo, n (%)*	270 (19.2%)	131 (16.5%)	139 (22.6%)	<0.001
*24–35 mo, n (%)*	404 (28.7%)	195 (24.6%)	209 (34.0%)	
*36–47 mo, n (%)*	330 (23.4%)	197 (24.8%)	133 (21.7%)	
*48–59 mo, n (%)*	404 (28.7%)	271 (34.1%)	133 (21.7%)	
Anthropometric data, x̄ (SD)				
*Child MUAC in cm*	14.73 (1.09)	14.58 (1.04)	14.91 (1.26)	<0.001
*Weight in kg*	12.94 (2.57)	13.33 (2.54)	12.43 (2.53)	<0.001
*Height in cm*	93.78 (10.88)	96.50 (10.46)	90.26 (10.41)	<0.001
Z-scores, x̄ (SD)				
*WHZ*	−0.84 (1.10)	−1.09 (1.07)	−0.53 (1.06)	<0.001
*HAZ*	−0.36 (1.51)	0.026 (1.40)	−0.85 (1.52)	<0.001
*MUACZ*	−0.76 (0.90)	−0.95 (0.86)	−0.53 (0.87)	<0.001
Stunting prevalence, n (%)				
*Not stunted (HAZ≥−2)*	1243 (88.3%)	744 (93.7%)	499 (81.3%)	<0.001
*Stunted (HAZ<−2)*	165 (11.7%)	50 (6.3%)	115 (18.7%)	
Wasting prevalences by Indicator, n (%) (95% CI)				
*MUAC<12.5cm*	21 (1.5%), (1.0, 2.3%)	14 (1.8%), (1.1, 3.0%)	7 (1.1%), (0.6, 2.4%)	0.17
*WHZ<−2*	208 (14.8%), 13.0, 16.7%)	161 (20.3%), (17.6, 23.2%)	47 (7.7%), (5.8, 10.1%)	<0.001
*Oedema*	4 (0.3%), (0.1, 0.8%)	2 (0.3%), (0.1,1.0%)	2 (0.3%), (0.1, 1.3%)	0.60
*2013 WHO Wasting Guidelines: MUAC<12.5cm and/or WHZ<−2 and/or Oedema*	218 (15.5%), (13.7, 17.5%)	166 (20.9%), (18.2, 23.9%)	52 (8.5%), (6.5, 11.0%)	<0.001
*MUACZ<−2*	120 (8.5%), (7.2, 10.1%)	91 (11.5%), (9.4, 13.9%)	29 (4.7%), (3.3, 6.7%)	<0.001
*Wasting by any of the 4 Measures: (MUAC, WHZ, Oedema, or MUACZ)*	256 (18.2%), (16.3, 20.3%)	192 (24.2%), (21.4, 27.3%)	64 (10.4%), (8.2, 13.1%)	<0.001
Pearson correlations between indicator pairs, ρ				
*MUAC and WHZ*	0.5501	0.4761	0.6095	
*MUACZ and WHZ*	0.6458	0.5848	0.6771	
*MUAC and MUACZ*	0.8455	0.8444	0.8437	

CI – confidence interval, MUAC – mid upper arm circumference, MUACZ – mid upper arm circumference z-score, SD – standard deviation, WHZ – weight-for-height z-score, x̄ – mean

*For additional analyses, child age was stratified into four categories (9–23 mo, 24–35 mo, 36–47 mo, and 48–59 mo) to evaluate changes in optimal wasting thresholds per year of child development.

Distributions of child characteristics can be found in Figure S1, Panels B–C in the **Online Supplementary Document**. Twenty-one children were diagnosed as wasted by MUAC<12.5cm (1.5% of the sample), 208 children were diagnosed as wasted by WHZ<-2 (14.8%), four children had oedema (0.3%), and 218 unique children (15.5%) were identified as wasted according to the 2013 WHO guidelines.

Analysing characteristics of wasted children (Table S1, Panel D in the **Online Supplementary Document**), there was no statistically significant difference in MUAC<12.5 cm wasting prevalence by child sex: 1.7% in male children *vs*. 1.3% in female children (*P* = 0.49). Male children (mean WHZ = −0.93, 95% CI = −1.01, −0.85) had a significantly lower mean WHZ than female children (mean WHZ = −0.76, 95% CI = −0.84, −0.68, *P* = 0.003). Male children also had a significantly higher wasting prevalence by WHZ<−2 (prevalence = 16.9%, 95% CI = 14.10, 19.65%) than female children (prevalence = 12.7%, 95% CI = 10.24, 15.14%, *P* = 0.03). Analysing wasting by age group (Table S1, Panel E in the **Online Supplementary Document**), 4.1% (95% CI = 1.72, 6.43%) of younger children, ages 9–23 months, were wasted by MUAC<12.5cm compared to 0.9% of older children, ages 24–59 months (95% CI = 0.34, 1.42%, *P* < 0.001). Older children had on average lower WHZ (mean WHZ = −0.89) than younger children (mean WHZ = −0.65, *P* < 0.001), but there was no statistically significant difference in wasting prevalence by WHZ<−2 between younger children (12.2%) and older children (15.4%, *P* = 0.12). The total wasting prevalence using the 2013 WHO guidelines was significantly different between regions (*P* < 0.001): 8.5% in Bay (95% CI = 6.27, 10.67%; 52 out of 614 children) and 20.9% in Hiran (95% CI = 18.08, 23.74%; 166 out of 794 children) (Table S5, Panel A in the **Online Supplementary Document**).

When using MUACZ<-2 as an alternative wasting indicator, 120 children were diagnosed as wasted by MUACZ alone (8.5%). Using any of the four indicators (MUAC<12.5cm, WHZ<−2, oedema, or MUACZ<−2), 256 unique children were identified as wasted, yielding a prevalence of 18.2% (95% CI = 16.25, 20.29%). Although the prevalence of wasting including MUACZ<−2 identified 38 additional children as wasted, this was not significantly different from the wasting prevalence by WHO guidelines (*P* = 0.06).

### Linear regression modelling

To explore relationships between WHZ and key anthropometric variables, linear regression was performed using WHZ as the outcome and MUAC, MUACZ, sex, age, stunting, and region as covariates. Table S3, Panel A in the **Online Supplementary Document** presents the regression modelling results.

In a simple linear regression of WHZ on continuous MUAC in Model 1, each cm change in MUAC was associated with a 0.56-unit increase in child WHZ (*P* < 0.001, R^2^ = 0.30), and a MUAC of 12.5cm was associated with a WHZ of −2.08. Simple linear regression of WHZ on additional covariates, age (continuous), sex, and stunting (yes/no), warranted their inclusion in a multiple linear regression as statistically significant covariates. Model 2 included both MUAC and age, showing age as a potential confounder of the relationship between MUAC and WHZ, resulting in a 17.4% change in the regression coefficient. Model 3 included all covariates with age as a continuous variable, and after adjustment, a MUAC = 12.5 cm was associated with WHZ = −1.46. Model 4 demonstrated no evidence of interaction between age and sex in the adjusted model. In Model 5, when region (Bay *vs*. Hiran) was added, the region coefficient was statistically significant (*P* = 0.003) but did not confound relationship between WHZ and any other covariates.

Linear regression Models 6 and 7 examined MUACZ. In Model 6, a 1-unit change in MUACZ was associated with a 0.788-unit change in WHZ, and a MUACZ = −2 was associated with WHZ = −1.82 (*P* < 0.001, R^2^ = 0.42). In the adjusted Model 7, age (*P* = 0.89) and sex (*P* = 0.12) were no longer statistically significant contributions, although stunting remained statistically significant (*P* < 0.001).

### Cohen’s Kappa and concordance between indicator pairs

Concordance values for each pair can be found in Table S4, Panel A in the **Online Supplementary Document**. MUAC<12.5cm and WHZ<2 together identified 216 unique children as wasted: 195 by WHZ only, 8 by MUAC<12.5cm only, and 13 by both MUAC<12.5cm and WHZ<−2 (Table S4, Panel E in the **Online Supplementary Document**). Treating WHZ<−2 as a ‘reference standard’ since it identifies more children as wasted, MUAC<12.5cm has a sensitivity of 6.25% and a specificity of 99.33%. Cohen’s kappa for concordance between MUAC<12.5 and WHZ<−2 was 0.089, indicating only slight concordance between these two wasting indicators. When stratifying the wasted group into MAM and SAM, MUAC identified 0 children as SAM and 21 children as MAM; WHZ identified 24 children as SAM and 184 children as MAM (Table S4, Panel F in the **Online Supplementary Document**).

MUAC<12.5 cm and MUACZ<−2 together identified 121 unique children as wasted: 100 by MUACZ<−2 only, 1 by MUAC<12.5 cm only, and 20 by both indicators (Table S4, Panel G in the **Online Supplementary Document**). Treating MUACZ<−2 as a reference standard, since it identifies more children as wasted, MUAC<12.5 cm has a sensitivity of 16.67% and a specificity of 99.92%. Cohen’s kappa for concordance between MUAC<12.5cm and MUACZ<−2 is 0.265, indicating fair concordance between these two wasting indicators. After stratifying the wasting group into MAM and SAM, MUACZ identified 10 children as SAM and 110 children as MAM (Table S4, Panel H in the **Online Supplementary Document**).

MUACZ<−2 and WHZ<-2 together identified 254 unique children as wasted: 134 by WHZ<−2 only, 46 by MUACZ<−2 only, and 74 by both indicators (Table S4, Panel I in the **Online Supplementary Document**). Treating WHZ<−2 as a reference standard, MUACZ<−2 has a sensitivity of 35.58% and a specificity of 96.17%. Cohen’s kappa for concordance between MUACZ<−2 and WHZ<−2 was 0.385, indicating fair concordance.

Stratifying the analysis by age group led to changes in concordance between each pair of wasting indicators (Table S4, Panel D in the **Online Supplementary Document**). Among children 9–23 months, Cohen’s kappa for MUAC and WHZ = 0.225 (fair concordance), MUAC and MUACZ = 0.758 (substantial concordance), and MUACZ and WHZ = 0.278 (fair concordance). Among children 24–59 months, Cohen’s kappa for MUAC and WHZ = 0.060 (slight concordance), MUAC and MUACZ = 0.160 (fair concordance), and MUACZ and WHZ = 0.403 (moderate concordance).

### Single-point ROC analysis and threshold determination

Single-point ROC analysis allowed us to compare current MUAC and MUACZ thresholds to a more ideal threshold that would improve their sensitivity for diagnosing wasting, using WHZ as the reference standard. ‘Optimal’ thresholds maximised AUC and sensitivity, while maintaining a false positive rate less than 35% (specificity >65%). Full sample ROC analysis results are found in **Table 2**.

**Table 2:** Full sample single-point ROC analysis results using WHZ as a reference standard

Full sample (n = 1408)			
MUAC	Sensitivity	Specificity	AUC
*MUAC<12.5cm*	6.3%	99.3%	0.528
*MUAC<14.4cm*	79.8%	68.3%	0.741
MUACZ	Sensitivity	Specificity	AUC
*MUACZ<−2*	35.6%	96.2%	0.659
*MUACZ<−1*	84.1%	68.0%	0.761

AUC – area under the curve, MUAC – mid upper arm circumference, MUACZ – mid upper arm circumference z-score, ROC – receiver operating characteristic, WHZ – weight-for-height z-score

For the entire study sample, the current threshold, MUAC<12.5 cm, yielded sensitivity = 6.3%, specificity = 99.3%, and AUC = 0.528, indicating discrimination between cases and non-cases of WHZ<−2 wasting that is only slightly better than by chance alone. The optimal MUAC threshold for the sample was 14.4 cm, yielding a sensitivity = 79.8%, specificity = 68.3%, and AUC = 0.741. For MUACZ<−2, sensitivity = 35.6%, specificity = 96.2%, and AUC = 0.659. The optimal MUACZ threshold for the sample was −1, yielding sensitivity = 84.1%, specificity = 68.0%, and AUC = 0.761. Given the difference in wasting prevalence between the Bay and Hiran regions of the study, we stratified the analysis to examine potential differences in the recommended MUAC or MUACZ threshold by region, but there were not major differences in optimal cutoffs by region (Table S5, Panels A–B in the **Online Supplementary Document**).

We also conducted ROC analysis stratifying by age at 24 months (Tables S6, Panels A–B in the **Online Supplementary Document**). Among children ages 9–23 months, MUAC<12.5 cm produces sensitivity = 18.2%, specificity = 97.9%, and AUC = 0.580. The optimal threshold for children ages 9–23 months was MUAC<13.7 cm, yielding sensitivity = 81.8%, specificity = 73.4%, and AUC = 0.774. Among children ages 24–59 months, MUAC<12.5 cm produces sensitivity = 4.0%, specificity = 99.7%, and AUC = 0.518. The optimal threshold for children ages 24–59 months was MUAC<14.4 cm, yielding sensitivity = 76.0%, specificity = 73.8%, and AUC = 0.749.

Among children ages 9–23 months, MUACZ<−2 produced sensitivity = 18.2%, specificity = 97.9%, and AUC = 0.580. The optimal threshold for children ages 9–23 months was MUACZ<−0.9, yielding sensitivity = 84.9%, specificity = 75.7%, and AUC = 0.802. Among children ages 24–59 months, MUACZ<−2 produced sensitivity = 37.7%, specificity = 96.0%, and AUC = 0.668. The optimal threshold for children ages 24–59 months was MUACZ<−1, yielding sensitivity = 85.1%, specificity = 65.3%, and AUC = 0.752.

ROC analysis was also stratified by age into four categories: 9–23 months, 24–35 months, 36–47 months, and 48–59 months. Optimal MUAC and MUACZ thresholds for each age category and performance results relative to WHZ are found in **Table 3**.

**Table 3:** Optimal MUAC & MUACZ thresholds, sensitivity, specificity for age-stratified analyses using WHZ as a reference standard

Age category	Optimal MUAC threshold	Sensitivity	Specificity	AUC
9–23 mo	MUAC<13.7 cm	81.82%	73.42%	0.776
24–35 mo	MUAC<14.4 cm	83.33%	65.19%	0.743
36–47 mo	MUAC<14.3 cm	73.33%	79.30%	0.763
48–59 mo	MUAC<14.4 cm	80.68%	71.30%	0.759
**Age category**	Optimal MUACZ threshold	Sensitivity	Specificity	AUC
9–23 mo	MUACZ<−0.9	84.85%	75.53%	0.802
24–35 mo	MUACZ<−1	73.81%	72.38%	0.731
36–47 mo	MUACZ<−1	84.44%	68.07%	0.763
48–59 mo	MUACZ<−1.4	79.55%	73.42%	0.765

AUC – area under the curve, MUAC – mid upper arm circumference, MUACZ – mid upper arm circumference z-score, WHZ – weight-for-height z-score

Since sex was a significant predictor of WHZ in regression models, we also conducted sex-stratified analyses to examine its differential impact on malnutrition diagnostics. Sex stratified results may be found in Table S7, Panels A–B in the **Online Supplementary Document**. While there were differences in WHZ<−2 wasting prevalence by child sex (12.7% in females, 16.9% in males; *P* = 0.027), the optimal MUAC threshold for both groups was 14.4 cm. In females, MUAC<14.4 cm produced sensitivity = 78.9%, specificity = 68.2%, AUC = 0.735, and in males, MUAC<14.4 cm produced sensitivity 80.5%, specificity = 68.5%, AUC = 0.745. MUACZ yielded a similar result, with optimal threshold of −1 for both females (sensitivity = 85.6%, specificity = 69.8%, AUC = 0.777) and males (sensitivity = 83.0%, specificity = 66.1%, AUC = 0.746).

## DISCUSSION

To our knowledge, this analysis is the first comparative assessment of MUAC, MUACZ, and WHZ diagnostic performance in two high wasting burden regions of Somalia. Our results indicate that the current MUAC<12.5cm to diagnose wasting heavily underestimates the number of children diagnosed as wasted by WHZ<−2, yielding a prevalence difference of 13 percentage points. MUAC<12.5 cm alone failed to diagnose 94% of the total WHZ<−2 wasted children, missing 100% of children SAM by WHZ (n = 24) and 94% of children MAM by WHZ (n = 208). This poor sensitivity of MUAC for diagnosing WHZ-wasted children aligns with studies conducted in other humanitarian settings with high malnutrition burden who similarly use MUAC only screening, including Cambodia, Ethiopia, South Sudan, and Guatemala [14,16–20]. Despite variations in body composition globally which can impact WHZ measurements, these studies agree that MUAC and WHZ diagnose wasting in different children [12].

In addition, minimal concordance (κ = 0.089) between current MUAC<12.5 cm and WHZ<−2 cutoffs shows that these two indicators diagnose different children as wasted: male children were more likely to be diagnosed as wasted by WHZ<−2, while younger children (ages 9–23 months) were more likely to be diagnosed as wasted by MUAC<12.5 cm. The poor concordance between MUAC and WHZ in this analysis was significantly lower than previous studies, having found fair concordance, with kappa ranging from 0.21–0.35 [14,17,20]. These studies suggest that global or even regional differences in wasting prevalence and body composition may impact concordance results, however, stratifying this analysis by region indicated little regional impact on concordance despite significantly different wasting prevalences. Slight concordance between MUAC and WHZ in this sample highlights the independence of the two measures and suggests they cannot be used interchangeably for wasting diagnosis.

Twenty children (9.2% of the total wasted children using WHO guidelines) were identified as SAM by WHZ but had a MUAC measurement within ‘normal’ range (≥12.5 cm). Of the total wasted children using WHO guidelines, 175 (80.3%) were identified as MAM by WHZ but had a MUAC measurement within ‘normal’ range. This discrepancy is alarming, since these children may not be referred for wasting treatment outside of this study due to reliance on MUAC alone for screening in Somalia and other settings utilizing MUAC only for admission to therapeutic care programmes. The 20 children who are severely malnourished according to weight-for-height are at high risk for additional health complications or death, but their vulnerable status is not captured by their MUAC measurement alone. The current MUAC<12.5 cm cutoff’s poor sensitivity (6.25%) for identifying children wasted by WHZ<−2 is insufficient to be used as a sole diagnostic indicator. Previous research has evaluated the use of MUACZ<−2 as an alternative indicator to measure wasting, with disagreement about its programmatic utility [12,35]. We evaluated adjusted MUAC thresholds and MUACZ to consider feasible alternatives to WHZ.

MUAC regression models indicated that higher MUAC, female sex, and stunting were associated with greater WHZ; older age and male sex were associated with significantly lower WHZ, suggesting that male children and children ages 24–59 months may be at greater risk for low weight-for-height. Age confounded the relationship between MUAC and WHZ. Model fit improved with the inclusion of adjustment covariates (Table S3, Panel A in the **Online Supplementary Document**, Model 3, R^2^ = 0.4585), but less than half of the variability in WHZ was explained by its relationship with the other anthropometric indicators. MUACZ regression models showed a similar trend, as the age and sex adjustment inherent in MUACZ improved model fit (Table S3, Panel A in the **Online Supplementary Document**, Model 4, R^2^ = 0.4171), but again failed to explain much of the variability in WHZ within these children. While the ability of these variables to predict 40–45% of WHZ is not trivial, MUAC and MUACZ are insufficient to be used interchangeably with WHZ for the prediction of wasting in CU5.

While MUACZ and WHZ saw improved concordance (κ = 0.385, fair concordance) compared to the MUAC and WHZ comparison, the sensitivity of 35.58% is still low. Eight children (0.6% of the sample) were identified as SAM by WHZ but normal by MUACZ, and 126 children (8.9% of the sample) were identified as MAM by WHZ but normal by MUACZ. This gap of 134 children, though smaller than the 195-child gap between MUAC and WHZ, still underestimates the true wasting prevalence. These findings underscore concerns for the programmatic utility of MUACZ found in global survey analysis and in the REDAC study conducted in Guatemala [12,20]. MUACZ thresholds may need further adjustment to ensure more wasted children receive treatment. Further stratifying age into four categories (9–23 months, 24–35 months, 36–47 months, and 48–59 months) highlighted two years of age as a critical age point to recommend different MUAC cutoffs, in agreement with previous literature utilising the same cutoff [12,15,18,22]. The 48–59-month age group also saw the highest concordance between MUACZ and WHZ, indicating potential improved concordance between MUACZ and WHZ around ages 4–5 years. Stratified analyses by region and sex showed the same optimal MUAC and MUACZ thresholds across groups despite varying wasting prevalences, indicating that child age was the most important factor impacting discordance between MUAC and WHZ.

These results did not vary from midline to endline in the study, with only minor differences in concordance and adjusted MUAC thresholds. For children 9–23 months, the optimal MUAC threshold was 13.7 cm at both midline and endline (Tables S6, Panel B and S14, Panel B in the **Online Supplementary Document**, respectively). For children 24–59 months, the optimal MUAC threshold was 14.5 cm at midline *vs*. 14.4 cm at endline (Table S6, Panel B and S14, Panel B in the **Online Supplementary Document**, respectively). While recommended thresholds are slightly different, the underlying message is the same: raising thresholds for wasting may help identify and treat more truly wasted children in Somalia.

### Programmatic implications

Previous literature and this analysis raise several considerations for nutrition programmes in humanitarian contexts. The 13-percentage point wasting prevalence difference using MUAC *vs*. WHZ in this analysis aligns with previous literature noting Somalia as an outlier due to larger differences in wasting prevalences using MUAC and WHZ compared to other countries [13,19]. MUAC as a sole diagnostic criterion failed to accurately identify 95% of WHZ-wasted children in this analysis and missed 25–80% of children in previous literature [12,13,16–18]. Programme implementers and policy makers should be aware of the major limitations of using MUAC alone to diagnose wasting in CU5. If no action is taken, wasted children will remain untreated, keeping malnutrition high and vulnerable children at risk of mortality.

While rapidly incorporating WHZ into community screening may be an ideal way to diagnose wasting, the cost and complications of obtaining accurate weight and height measures in low-resource contexts may make this challenging. This study and previous evidence for updating wasting measurement guidelines prompt two alternative solutions for consideration:

1) updating the MUAC threshold and stratifying by age at 24 months to align more closely with a WHZ of −2,

2) or using the age-adjusted MUACZ as an alternative measurement.

Annex S20 in the **Online Supplementary Document** outlines the advantages, disadvantages, and implementation considerations of measures for diagnosing CU5 wasting during community screenings.

Utilizing an updated and age-stratified MUAC threshold in this analysis correctly identified 75–80% of WHZ-wasted children, yielding a significant improvement over MUAC<12.5 cm. An age-stratified MUAC threshold accounts for clear differences in child growth before and after 24 months and ensures that children aged 24–59 months, who are especially vulnerable to this diagnostic misclassification, are more effectively screened for wasting.

While the age-stratified MUAC threshold would improve accuracy in wasting diagnosis, there are programmatic and ethical barriers to implementation. Applying multiple MUAC cutoffs complicates the activities undertaken by community health workers, requiring additional training, increased supervision, and longer time for measurements and age-verification, which may not be feasible. In Somalia and similar settings, age-specific wasting guidelines may be also susceptible to inaccuracies in age data. In contexts with a high malnutrition burden, these additional tasks may reduce the number of children CHWs are able to screen each day. In addition to these workforce concerns, updating measurement guidelines would increase the number of children enrolled in wasting treatment programmes, subsequently increasing the supplies, health care workers, and infrastructure needed for treatment. These requirements will impact the distribution and availability of supplies and increase funding needs for treatment programmes with already limited resources. With increased caseloads and strained budgets, health care facilities and local governments would have to make decisions addressing effective and equitable resource allocation.

Alternatively, introducing MUACZ to screen for wasting may offer similar benefits in diagnostic accuracy. In this analysis, MUACZ<−2 identified 36% of WHZ-wasted children and already performed far better than MUAC<12.5 cm for wasting. This analysis suggested that even MUACZ thresholds should be adjusted to improve sensitivity, but updating wasting guidelines using MUACZ offers the same programmatic concerns for workforce, supply, and funding issues as the age-stratified MUAC thresholds. Since MUACZ is calculated based on child age in months, these measurements would be very susceptible to imprecise age data in Somalia and similar contexts without comprehensive vital registration data. This critical implementation barrier would require thorough and context-specific consideration before MUACZ could be adapted and scaled for programmatic use.

The technical difficulties of anthropometry can reduce measurement accuracy and lead to diagnostic errors, especially in young children. If alternative measures for wasting diagnosis are to be considered, programme implementers should take efforts to robustly train and retrain community health workers on best practices, while strengthening supervision and monitoring to maintain diagnostic accuracy.

It may be most feasible for Somalia to implement the solution of an increased MUAC threshold stratified by age at 24 months, rather than adopting new measurements. Yet, donors and nutrition stakeholders need to carefully consider how raising MUAC thresholds could impact health systems and health financing.

### Study limitations and strengths

As with all evaluations, there are limitations to this work. Some known limitations include a smaller data set than many previous studies using global anthropometric data; however, our sample size was over n = 1000, which is appropriately powered for these analyses. Stratified results must be interpreted with caution due to smaller sample sizes. The context changed from baseline to endline due to droughts, flooding, and displacement of study participants, which may have affected measurement rigor. Some participants were lost to follow up at endline, but the demographic distribution of the population remained consistent despite this loss. Vital registration data for child age in the study may be less accurate compared to other contexts. This exploratory study enrolled a healthy cohort at baseline (not wasted using MUAC) therefore wasting prevalence and concordance estimates in this analysis may be skewed, underestimating the true malnutrition burden in these regions. Broader surveillance efforts and large-scale surveys in Somalia are needed to better estimate wasting prevalence and measurement discordance in order to inform policy planning and treatment decision-making.

As with all studies collecting anthropometric data, measurement error and inter-observer variability may impact results. However, the research team addressed these concerns where possible by rigorously training all enumerators who collected survey and anthropometric data for the clinical trial. Enumerators utilised SMART methodology for standard anthropometric measurements and measured each child’s weight, height, and MUAC twice before recording.

Our study does have considerable strengths. Our large sample size was well-powered for advanced statistical analyses and stratification. Our sample had a significantly higher wasting prevalence than the global average of 6.8% and we produced timely wasting prevalences within 6 months of data collection. This study was conducted in a vulnerable context and directly identifies children who can benefit from more accurate screening measures for malnutrition. Our cluster randomised controlled trial may have more accurate and consistently measured anthropometric data than larger national survey data sets.

Future research into concordance between MUAC, MUACZ and WHZ in global contexts should assess how various MUAC and MUACZ thresholds improve sensitivity with WHZ. Future studies in Somalia should examine how well these recommended thresholds may perform across other regions and settings. Further studies should be conducted to assess the impact of adopting MUACZ and WHZ on workforce, supply, ease-of-use, and time requirements. Lastly, given the resource-intensive nature of implementing WHZ and MUACZ, additional costing analyses, such as Value for Money (VfM), should evaluate the financial implications and cost-effectiveness of scaling MUACZ or WHZ in low-resource settings.

## CONCLUSIONS

Our results demonstrate poor concordance between MUAC and WHZ for diagnosing wasting among CU5 in two high wasting burden regions of Somalia. Although MUACZ had improved concordance, it still heavily underestimated WHZ wasting prevalence. While WHZ may be a preferred measure for wasting diagnosis, it remains an expensive and resource-intensive diagnostic technique. Age-stratified MUAC thresholds which identify a much larger proportion of WHZ-wasted children provide an alternative, more feasible diagnostic recommendation for community screening of malnutrition. For wasting screening in CU5 to be most effective, the global community must acknowledge the limitations of current anthropometric measures for wasting. Particularly international partners, donors, and government stakeholders in Somalia should consider this evidence and evaluate revision of wasting guidelines to treat and prevent future wasting in vulnerable children.

## Additional material


Online Supplementary Document
