# Supplementary material for: Analysing concordance between MUAC, MUACZ, and WHZ in diagnosing acute malnutrition among children under five in Somalia
Source: J Glob Health. 2025 Dec 12;15:04258. doi: 10.7189/jogh.15.04258 (PMC12699285; doi:10.7189/jogh.15.04258)
Supplement: Online Supplementary Document [file jogh-15-04258-s001.pdf]

Supplement to: Garretson S, Walton S, Alier K, Grounds S, Khattak Q, Mohamoud S, Farah A, Mohamud F, Mohamoud A, Mahdi A, Ismail M, Mahat M, Loddo F, Tripaldi M, Akseer N. Analysing concordance between MUAC, MUACZ, and WHZ in diagnosing acute malnutrition among children under 5 in Somalia. J Glob Health. 2025;15:04258.

Supplemental Files S1-S20

Full Supplemental Files Table of Contents

Annex S1. Child Characteristics and Distributions - Endline
Table S1A. Acute Malnutrition Definitions, including MAM and SAM, by Anthropometric Indicator
Figure S1B. Distributions of Child Anthropometric Characteristics: Child Age, Height, MUAC, Weight
Figure S1C. Distributions of Child Anthropometric Characteristics: Z-Scores
Table S1D. Anthropometric Differences by Child Sex
Table S1E. Anthropometric Differences by Child Age (9-23 months vs. 24-59 months)
Annex S2. Pearson's Correlation Figures - Endline
Figure S2A. Pearson's Correlation between MUAC and WHZ
Figure S2B. Pearson's Correlation between MUAC and MUACZ
Figure S2C. Pearson's Correlation between MUACZ and WHZ
Annex S3. Linear Regression Modelling Results - Endline
Table S3A. Regression Modelling for Endline Sample
Annex S4. Concordance Results - Endline
Table S4A. Entire Sample (N=1408) Concordance Pairs: Wasted vs. Not Wasted
Table S4B. Entire Sample (N=1408) Concordance Pairs: Normal vs. MAM vs. SAM
Table S4C. Concordance Pairs Stratified by Region: Wasted vs. Not Wasted
Table S4D. Concordance Pairs Stratified by Age (9-23 months vs 24-59 months): Wasted vs. Not Wasted
Table S4E. Number of Children Diagnosed as Wasted Using MUAC and/or WHZ Criteria
Table S4F. Number of Children Diagnosed as Normal vs. MAM vs. SAM Using MUAC and/or WHZ Criteria
Table S4G. Number of Children Diagnosed as Wasted Using MUAC and/or MUACZ Criteria
Table S4H. Number of Children Diagnosed as Normal vs. MAM vs. SAM Using MUAC and/or MUACZ Criteria
Table S4I. Number of Children Diagnosed as Wasted Using MUACZ and/or WHZ Criteria
Table S4J. Number of Children Diagnosed as Normal vs. MAM vs. SAM Using MUACZ and/or WHZ Criteria
Annex S5. Stratified Analysis: by Region (Bay vs. Hiran) - Endline
Table S5A. Wasting Prevalence by Region
Table S5B. ROC Analysis by Region
Annex S6. Stratified Analysis: by Child Age (9-23 mo. vs 24-59 mo.) - Endline
Table S6A. Wasting Prevalence by Child Age
Table S6B. ROC Analysis by Child Age
Annex S7. Stratified Analysis: by Child Sex (Female vs. Male) – Endline
Table S7A. Wasting Prevalence by Child Sex
Table S7B. ROC Analysis by Child Sex

Table S8. Midline Analysis Demographics Table
Annex S9. Midline Analysis Child Characteristics and Distributions
Figure S9A. Distributions of Child Anthropometric Characteristics: Child Age, Height, MUAC, Weight
Figure S9B. Distributions of Child Anthropometric Characteristics: Z-Scores
Table S9C. Anthropometric Differences by Child Sex
Table S9D. Anthropometric Differences by Child Age (9-23 months vs. 24-59 months)
Annex S10. Midline Analysis Pearson's Correlation Figures
Figure S10A. Pearson's Correlation between MUAC and WHZ
Figure S10B. Pearson's Correlation between MUAC and MUACZ
Figure S10C. Pearson's Correlation between MUACZ and WHZ
Annex S11. Midline Analysis Linear Regression Modelling Results
Table S11A. Regression Modelling for Endline Sample
Annex S12. Midline Analysis Concordance Results
Table S12A. Entire Sample Concordance Pairs: Wasted vs. Not Wasted
Table S12B. Entire Sample Concordance Pairs: Normal vs. MAM vs. SAM
Table S12C. Concordance Pairs Stratified by Region: Wasted vs. Not Wasted
Table S12D. Concordance Pairs Stratified by Age (9-23 months vs 24-59 months): Wasted vs. Not Wasted
Table S12E. Number of Children Diagnosed as Wasted Using MUAC and/or WHZ Criteria
Table S12F. Number of Children Diagnosed as Normal vs. MAM vs. SAM Using MUAC and/or WHZ Criteria
Table S12G. Number of Children Diagnosed as Wasted Using MUAC and/or MUACZ Criteria
Table S12H. Number of Children Diagnosed as Normal vs. MAM vs. SAM Using MUAC and/or MUACZ Criteria
Table S12I. Number of Children Diagnosed as Wasted Using MUACZ and/or WHZ Criteria
Table S12J. Number of Children Diagnosed as Normal vs. MAM vs. SAM Using MUACZ and/or WHZ Criteria
Annex S13. Midline Full Sample ROC Analysis
Table S13A. Current MUAC Thresholds, Sensitivity, Specificity, AUC – Midline
Table S13B. Ideal MUAC Thresholds, Sensitivity, Specificity, AUC
Table S13C. Ideal MUAC Thresholds, Sensitivity, Specificity, AUC
Annex S14. Midline Stratified Analysis: by Region (Bay vs. Hiran)
Table S14A. Wasting Prevalence by Region
Table S14B. ROC Analysis by Region
Annex S15. Midline Stratified Analysis: by Child Age (9-23 mo. vs 24-59 mo.)
Table S15A. Wasting Prevalence by Child Age
Table S15B. ROC Analysis by Child Age
Annex S16. Midline Stratified Analysis: by Child Sex (Female vs. Male)
Table S16A. Wasting Prevalence by Child Sex
Table S16B. ROC Analysis by Child Sex
Annex S17. Description of Bay and Hiran Regions of Somalia
Annex S18. Detailed Description of CashPlus for Nutrition Study Protocol
Annex S19. Additional Methods: Analysis Approach
Annex S20. Comparison of Wasting Measurement Indicators in Practice

Annex S1. Child Characteristics and Distributions – Endline

Table S1A. Acute Malnutrition Definitions, including MAM and SAM, by Anthropometric Indicator

**Acute malnutrition measurement indicator and category**	**Clinical Cutoff**
2013 World Health Organization Guidelines	
Not wasted	MUAC ≥ 12.5cm and WHZ ≥ -2
Wasted	MUAC < 12.5cm and/or WHZ < -2 and/or edema
MAM	11.5cm ≤ MUAC < 12.5cm and/or -3≤ WHZ < -2, without edema
SAM	MUAC < 11.5cm and/or WHZ < -3 and/or with edema
Mid-upper arm circumference (MUAC)	
Not wasted	MUAC ≥ 12.5cm
Wasted	MUAC < 12.5cm
MAM	11.5 cm ≤ MUAC < 12.5cm
SAM	MUAC < 11.5cm
Weight-for-height z-score (WHZ)	
Not wasted	WHZ ≥ -2
Wasted	WHZ < -2
MAM	-3≤ WHZ< -2
SAM	WHZ < -3
Mid-upper arm circumference z-score (MUACZ)	
Not wasted	MUACZ ≥ -2
Wasted	MUACZ < -2
MAM	-3 ≤ MUACZ < -2
SAM	MUACZ < -3
Edema	
Not wasted	Without bilateral pitting edema
Wasted (MAM)	Without bilateral pitting edema
Wasted (SAM)	With bilateral pitting edema

MAM - Moderate Acute Malnutrition. SAM - Severe Acute Malnutrition. WHZ – weight-for-height z-score.

Figure S1B. Distributions of Child Anthropometric Characteristics: Child Age, Height, MUAC, Weight

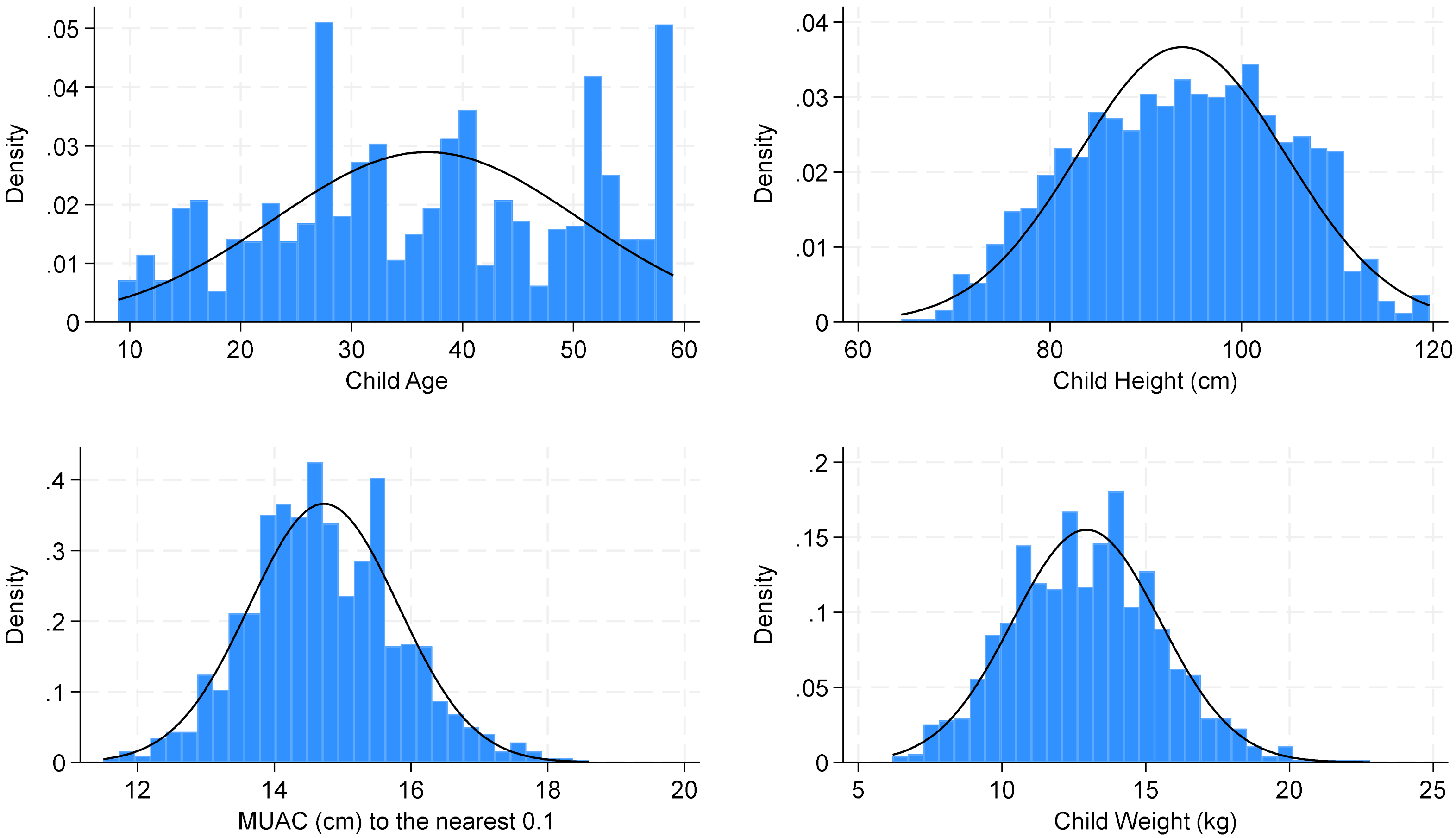

MUAC – mid upper arm circumference.

Figure S1C. Distributions of Child Anthropometric Characteristics: Z-Scores

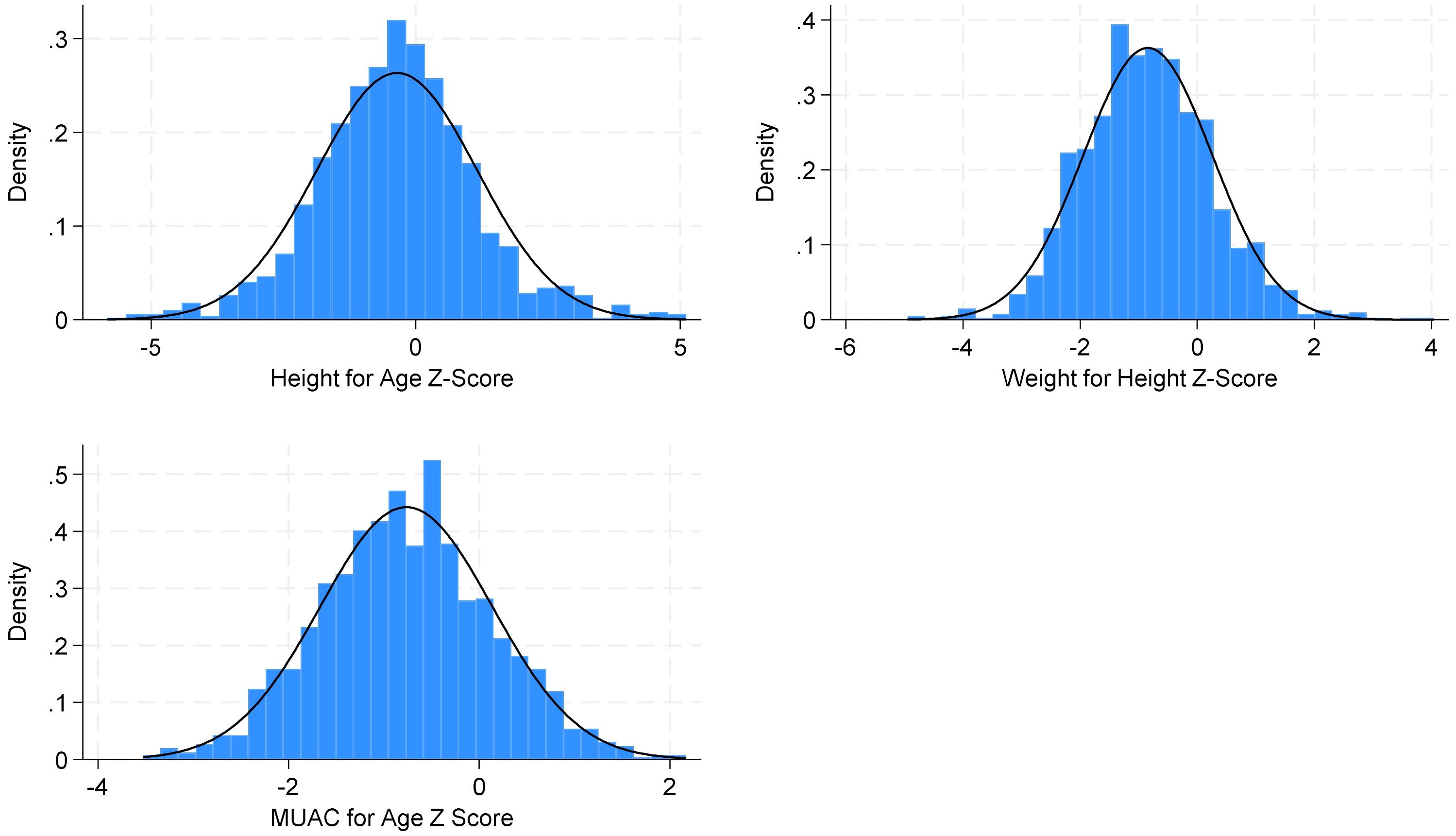

MUAC – mid upper arm circumference.

Table S1D. Anthropometric Differences by Child Sex

Anthropometric Measurement	Male (n=687)	Female (n=700)	p-value
Wasting Prevalence by MUAC	1.72% (n=12)	1.27% (n=9)	0.489
Mean MUAC (cm)	14.72cm	14.74cm	0.748
Wasting Prevalence by WHZ	16.88% (n=118)	12.69% (n=90)	0.027*
Mean WHZ	-0.93	-0.76	0.003*

MUAC – mid upper arm circumference. WHZ – weight-for-height z-score.
*p<0.05.

Table S1E. Anthropometric Differences by Child Age (9-23 months vs. 24-59 months)

Anthropometric Measurement	9-23 months (n=270)	24-59 months (n=1,138)	p-value
Wasting Prevalence by MUAC	4.07% (n=11)	0.88% (n=10)	<0.001*
Mean MUAC (cm)	14.18cm	14.86cm	<0.001*
Wasting Prevalence by WHZ	12.22% (n=33)	15.38% (n=175)	0.189
Mean WHZ	-0.65	-0.89	0.002*

MUAC – mid upper arm circumference. WHZ – weight-for-height z-score.
*p<0.05.

Annex S2. Pearson’s Correlation Figures - Endline

Figure S2A. Pearson’s Correlation between MUAC and WHZ: ρ=0.5501

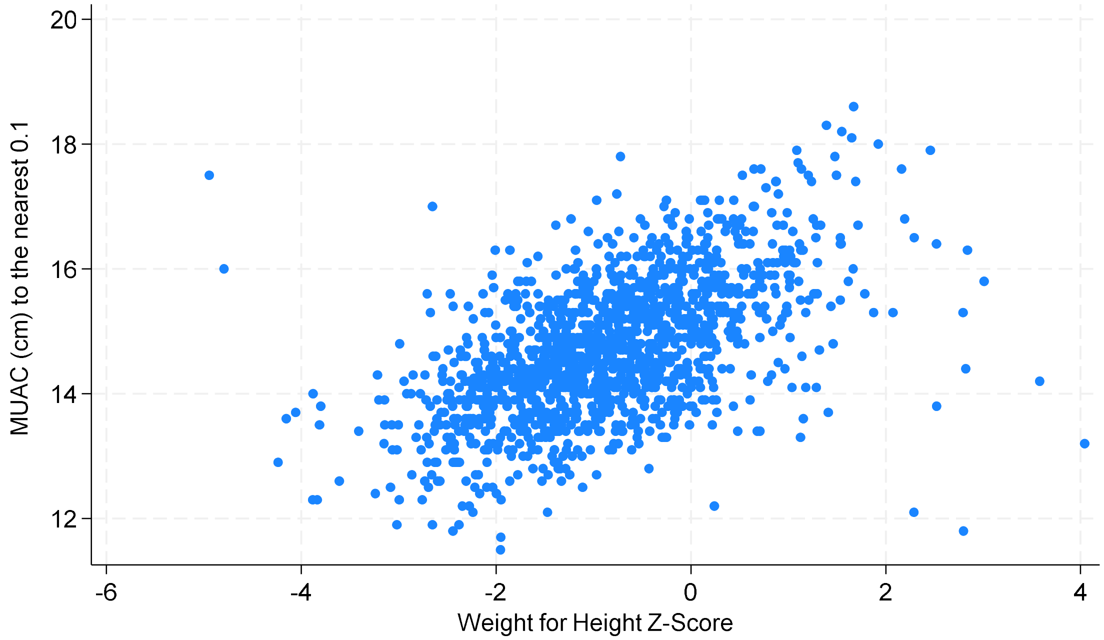

MUAC – mid upper arm circumference. WHZ – weight-for-height z-score.

Figure S2B. Pearson's Correlation between MUAC and MUACZ: ρ=0.8455

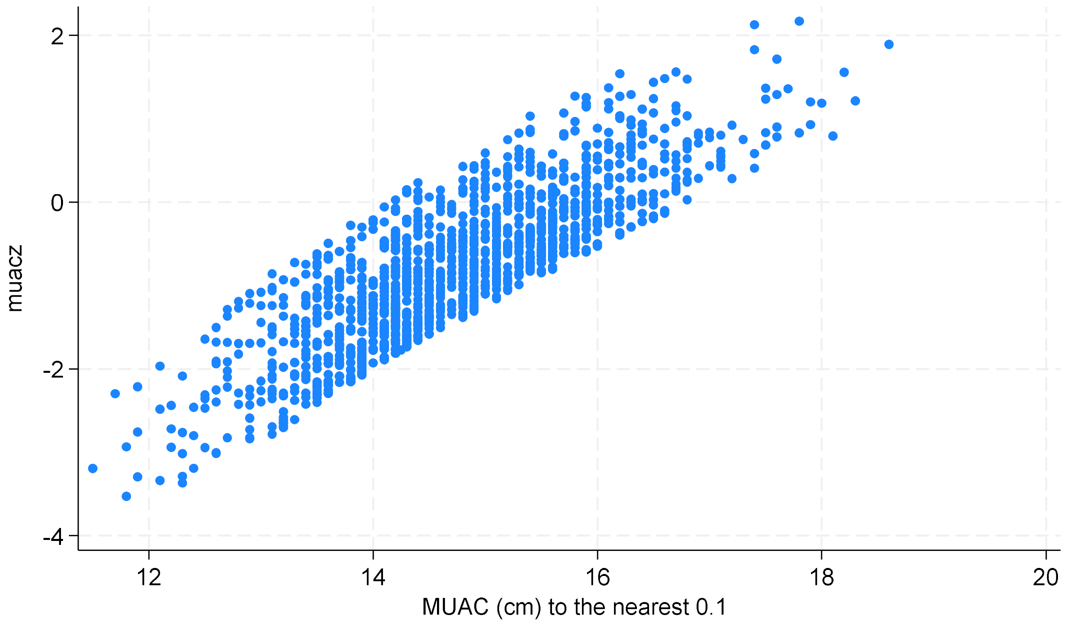

MUAC – mid upper arm circumference. MUACZ – mid upper arm circumference z-score. WHZ – weight-for-height z-score.

Figure S2C. Pearson's Correlation between MUACZ and WHZ: ρ=0.6458

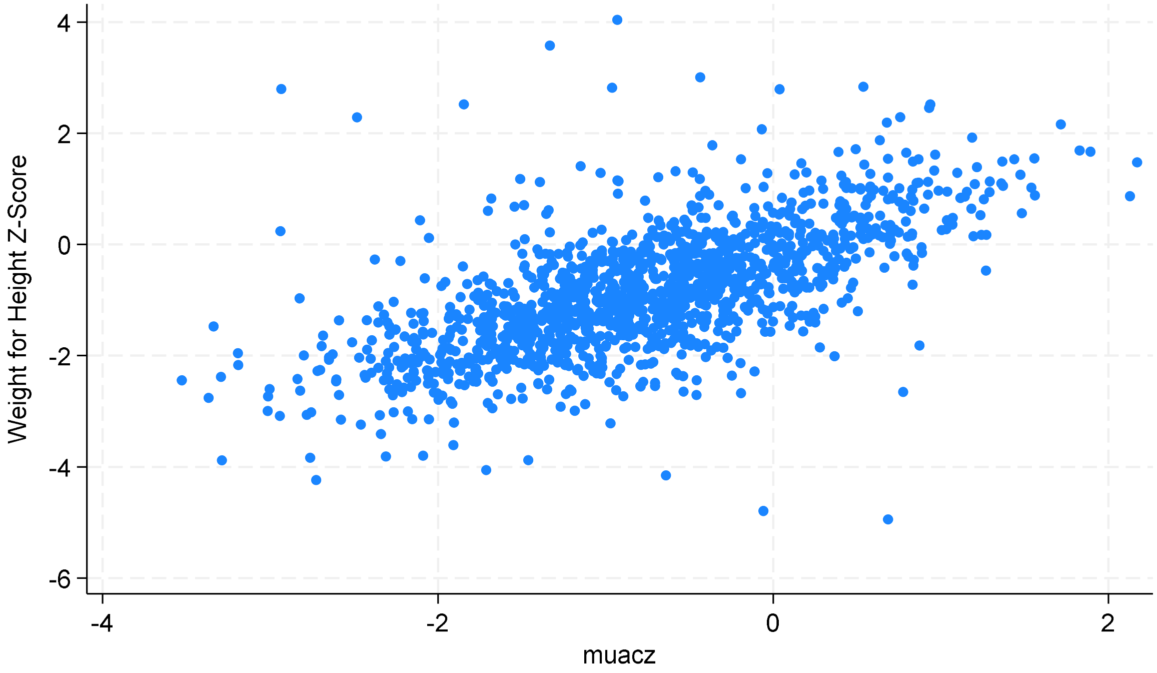

MUACZ – mid upper arm circumference z-score. WHZ – weight-for-height z-score.

Annex S3. Regression Modelling – Endline

Table S3A. Regression Modelling for Endline Sample

	**Model 1** β (95% CI)	**Model 2** β (95% CI)	**Model 3** β (95% CI)	**Model 4** β (95% CI)	**Model 5** β (95% CI)	**Model 6** β (95% CI)	**Model 7** β (95% CI)	**Model 8** β (95% CI)
MUAC	0.56‡ (0.50, 0.61)	0.65‡ (0.61, 0.70)	0.67‡ (0.63, 0.72)	0.68‡ (0.63, 0.72)	0.66‡ (0.61, 0.71)	-	-	0.67‡ (0.62,0.71)
Age (continuous)	-	-0.03‡ (-0.03, -0.02)	-0.03‡ (-0.03, -0.03)	-0.03‡ (-0.03, -0.02)	-0.03‡ (-0.03, -0.02)	-	0.0002 (-0.004, 0.004)	-
Sex (Female)	-	-	0.16‡ (0.07, 0.24)	0.21* (0.04, 0.38)	0.15‡ (0.07, 0.24)	-	0.07 (-0.02, 0.15)	0.16‡ (0.07, 0.24)
Stunting (Stunted vs. not stunted)	-	-	0.65‡ (0.50, 0.79)	0.65‡ (0.50, 0.79)	0.60‡ (0.46, 0.75)	-	0.66‡ (0.52, 0.81)	0.65‡ (0.50, 0.79)
Age * Sex	-	-	-	-0.06 (-0.24, 0.12)	-	-	-	-
Region (Bay vs Hiran)	-	-	-	-	0.14‡ (0.05, 0.22)	-	-	-
MUACZ	-	-	-	-	-	0.79‡ (0.73, 0.84)	0.81‡ (0.75, 0.87)	-
Age (reference category 9-23 months)	-	-	-	-	-	-	-	-
24-35 months	-	-	-	-	-	-	-	-0.38‡ (-0.52, -0.25)
36-47 months	-	-	-	-	-	-	-	-0.69‡ (-0.82, -0.55)
48-59 months	-	-	-	-	-	-	-	-1.04‡ (-1.16, -0.91)
**Model R^2**	**0.3026**	**0.4176**	**0.4585**	**0.4587**	**0.4618**	**0.4171**	**0.4554**	**0.4489**

CI – confidence interval. MUAC – mid upper arm circumference. MUACZ – mid upper arm circumference z-score. WHZ – weight-for-height z-score.

*p<0.05. †p<0.01. ‡p<0.001.

Note: MUAC and MUACZ were modelled as continuous variables. Sex, stunting, and region were modelled as dichotomous variables. Age was modelled as both a continuous variable and a categorical variable, depending on the model. Models met assumptions for normality, linearity, independence, and non-multicollinearity. Robust variance was used for all models.

Note on Model 8: Linear combinations for this regression model indicated that all categories were statistically significant from each other and all p<0.001.

Annex S4. Tables with Concordance Pairs - Endline

Table S4A. Entire Sample (N=1408) Concordance Pairs: Wasted vs. Not Wasted

Pair of Wasting Indicators	Kappa	Strength of Concordance
MUAC & WHZ	0.0889	None-slight
MUAC & MUACZ	0.2650	Fair
MUACZ & WHZ	0.3847	Fair (almost moderate)

MAM – moderate acute malnutrition. MUAC – mid upper arm circumference. MUACZ – mid upper arm circumference z-score. WHZ – weight-for-height z-score. SAM – severe acute malnutrition.

Table S4B. Entire Sample (N=1408) Concordance Pairs: Normal vs. MAM vs. SAM

Pair of Wasting Indicators	Kappa	Strength of Concordance
MUAC & WHZ	0.0724	None-slight
MUAC & MUACZ	0.2077	Fair
MUACZ & WHZ	0.3169	Fair

MAM – moderate acute malnutrition. MUAC – mid upper arm circumference. MUACZ – mid upper arm circumference z-score. WHZ – weight-for-height z-score. SAM – severe acute malnutrition.

Table S4C. Concordance Pairs Stratified by Region: Wasted vs. Not Wasted

Pair of Wasting Indicators	Bay Kappa	Hiran Kappa	Total
MUAC & WHZ	0.0931	0.0846	0.0889
MUAC & MUACZ	0.3209	0.2435	0.2650
MUACZ & WHZ	0.2928	0.1761	0.3847

MAM – moderate acute malnutrition. MUAC – mid upper arm circumference. MUACZ – mid upper arm circumference z-score. WHZ – weight-for-height z-score. SAM – severe acute malnutrition.

Table S4D. Concordance Pairs Stratified by Age (9-23 months vs 24-59 months): Wasted vs. Not Wasted

Pair of Wasting Indicators	9-23 months Kappa	24-59 months Kappa	Total
MUAC & WHZ	0.2254	0.06	0.0889
MUAC & MUACZ	0.7578	0.1604	0.2650
MUACZ & WHZ	0.2782	0.4025	0.3847

MAM – moderate acute malnutrition. MUAC – mid upper arm circumference. MUACZ – mid upper arm circumference z-score. WHZ – weight-for-height z-score. SAM – severe acute malnutrition.

Table S4E. Number of Children Diagnosed as Wasted Using MUAC and/or WHZ Criteria

		Child Wasting by WHZ		
		Not wasted	Wasted	**Total**
**Child Wasting by MUAC**	Not wasted	1192	195	1387
	Wasted	8	13	21
	**Total**	1200	208	1408

MAM – moderate acute malnutrition. MUAC – mid upper arm circumference. MUACZ – mid upper arm circumference z-score. WHZ – weight-for-height z-score. SAM – severe acute malnutrition.

Table S4F. Number of Children Diagnosed as Normal vs. MAM vs. SAM Using MUAC and/or WHZ Criteria

		Child Wasting by WHZ			
		SAM	MAM	Normal	**Total**
**Child Wasting by MUAC**	SAM*	0	0	0	0
	MAM	4	9	8	21
	Normal	20	175	1192	1387
	**Total**	24	184	1200	1408

MAM – moderate acute malnutrition. MUAC – mid upper arm circumference. MUACZ – mid upper arm circumference z-score. WHZ – weight-for-height z-score. SAM – severe acute malnutrition.

*Note: No children were diagnosed as SAM by MUAC (<11.5cm)

Table S4G. Number of Children Diagnosed as Wasted Using MUAC and/or MUACZ Criteria

		Child Wasting by MUACZ		
		Not wasted	Wasted	**Total**
**Child Wasting by MUAC**	Not wasted	1287	100	1387
	Wasted	1	20	21
	**Total**	1288	120	1408

MAM – moderate acute malnutrition. MUAC – mid upper arm circumference. MUACZ – mid upper arm circumference z-score. WHZ – weight-for-height z-score. SAM – severe acute malnutrition.

Table S4H. Number of Children Diagnosed as Normal vs. MAM vs. SAM Using MUAC and/or MUACZ Criteria

		Child Wasting by MUACZ			
		SAM	MAM	Normal	**Total**
**Child Wasting by MUAC**	SAM	0	0	0	0
	MAM	8	12	1	21
	Normal	2	98	1287	1387
	**Total**	10	110	1288	1408

MAM – moderate acute malnutrition. MUAC – mid upper arm circumference. MUACZ – mid upper arm circumference z-score. WHZ – weight-for-height z-score. SAM – severe acute malnutrition.

Table S4I. Number of Children Diagnosed as Wasted Using MUACZ and/or WHZ Criteria

		Child Wasting by WHZ		
		Not wasted	Wasted	**Total**
**Child Wasting by MUACZ**	Not wasted	1154	134	1288
	Wasted	46	74	120
	**Total**	1200	208	1408

MAM – moderate acute malnutrition. MUAC – mid upper arm circumference. MUACZ – mid upper arm circumference z-score. WHZ – weight-for-height z-score. SAM – severe acute malnutrition.

Table S4J. Number of Children Diagnosed as Normal vs. MAM vs. SAM Using MUACZ and/or WHZ Criteria

		Child Wasting by WHZ			
		SAM	MAM	Normal	**Total**
**Child Wasting by MUACZ**	SAM	1	7	2	10
	MAM	15	51	44	110
	Normal	8	126	1154	1288
	**Total**	24	184	1200	1408

MAM – moderate acute malnutrition. MUAC – mid upper arm circumference. MUACZ – mid upper arm circumference z-score. WHZ – weight-for-height z-score. SAM – severe acute malnutrition.

Annex S5. Stratified Analysis by Region (Bay vs. Hiran) - Endline

Table S5A. Wasting Prevalence by Region (Bay vs. Hiran)

**Indicator**	**Bay** (n=614)	**Hiran** (n=794)	**p-values**
MUAC	**1.14%** (n=7)<br>(95% CI: 0.54%, 2.38%)	**1.76%** (n=14)<br>(95% CI: 1.05%, 2.96%)	0.339
WHZ	**7.65%** (n=47)<br>(95% CI: 5.80%, 10.05%)	**20.28%** (n=161)<br>(95% CI: 17.62%, 23.22%)	<0.001*
Edema	**0.33%** (n=2)<br>(95% CI: 0.08%, 1.30%)	**0.25%** (n=2)<br>(95% CI: 0.06%, 1.00%)	0.796
WHO Definition Wasted:<br>by MUAC or WHZ or Edema	**8.47%** (n=52)<br>(95% CI: 6.51%, 10.95%)	**20.91%** (n=166)<br>(95% CI: 18.21%, 23.88%)	<0.001*
MUACZ	**4.72%** (n=29)<br>(95% CI: 3.30%, 6.72%)	**11.46%** (n=91)<br>(95% CI: 9.42%, 13.87%)	<0.001*
Wasting by all 4 Measures:<br>by MUAC, WHZ, eedema, or MUACZ	**10.42%** (n=64)<br>(95% CI: 8.24%, 13.11%)	**24.18%** (n=192)<br>(95% CI: 21.35%, 27.29%)	<0.001*

CI – confidence interval. MUAC – mid upper arm circumference. MUACZ – mid upper arm circumference z-score. WHZ – weight-for-height z-score.
*p<0.05

Table S5B. ROC Analysis by Region (Bay vs. Hiran)

**ROC Analysis Results**	**Bay**	**Hiran**
Ideal MUAC Threshold	14.4cm	14.3cm
MUAC AUC	0.8012	0.7123
MUAC Sensitivity	87.23%	74.53%
MUAC Specificity	73.02%	67.93%
Ideal MUACZ Threshold	-0.9	-1.1
MUACZ AUC	0.7800	0.7308
MUACZ Sensitivity	85.11%	79.50%
MUACZ Specificity	70.90%	66.67%

ROC- receiver operating characteristic. MUAC – mid upper arm circumference. MUACZ – mid upper arm circumference z-score. WHZ – weight-for-height z-score. AUC – area under the curve.
Note: There was minimal difference in the ideal MUAC by region, but the sensitivities, specificities, and AUCs are very different, but this is likely due to differences in age or stunting prevalence by region. Similarly to MUAC, there was minimal difference in the ideal MUACZ by region, but the sensitivities, specificities, and AUCs are different.

Annex S6. Stratified Analysis by Child Age (9-23 months vs 24-59 months) - Endline

Table S6A. Wasting Prevalences by Child Age (9-23 months vs 24-59 months)

**Indicator**	**9-23 months** (n=273)	**24-59 months** (n=1,138)	**p-values**
MUAC	**4.07%** (n=11)<br>(95% CI: 2.26%, 7.22%)	**0.88%** (n=10)<br>(95% CI: 0.47%, 1.63%)	<0.001*
WHZ	**12.22%** (n=33)<br>(95% CI: 8.81%, 16.72%)	**15.38%** (n=175)<br>(95% CI: 13.39%, 17.59%)	0.189
Edema	**0.37%** (n=1)<br>(95% CI: 0.05%, 2.6%)	**0.26%** (n=3)<br>(95% CI: 0.08%, 0.82%)	0.767
WHO Definition Wasted: by MUAC **or** WHZ **or** Edema	**14.07%** (n=38)<br>(95% CI: 10.40%, 18.78%)	**15.82%** (n=180)<br>(95% CI: 13.81%, 18.06%)	0.477
MUACZ	**5.56%** (n=15)<br>(95% CI: 3.37%, 9.03%)	**9.23%** (n=105)<br>(95% CI: 7.68%, 11.05%)	0.052
Wasting by all 4 Measures: (MUAC, WHZ, Edema, MUACZ)	**14.81%** (n=40)<br>(95% CI: 11.04%, 19.59%)	**18.98%** (n=216)<br>(95% CI: 16.80%, 21.37%)	0.111

CI – confidence interval. MUAC – mid upper arm circumference. MUACZ – mid upper arm circumference z-score. WHZ – weight-for-height z-score.
*$p<0.05$ **Note:** There was a borderline significant difference in wasting prevalence by MUACZ between the age groups. These differences should be interpreted with caution due to the difference in sample sizes between the two age groups and small size of the 9-23 months old group.

Table S6B. ROC Analysis by Child Age (9-23 months vs 24-59 months)

**ROC Analysis Results**	**9-23 months**	**24.59 months**
Ideal MUAC Threshold	13.7cm	14.4cm
MUAC AUC	0.7762	0.7492
MUAC Sensitivity	81.82%	76.00%
MUAC Specificity	73.42%	73.83%
Ideal MUACZ Threshold	-0.9	-1
MUACZ AUC	0.8019	0.7523
MUACZ Sensitivity	84.85%	85.14%
MUACZ Specificity	75.53%	65.32%

ROC- receiver operating characteristic. MUAC – mid upper arm circumference. MUACZ – mid upper arm circumference z-score. WHZ – weight-for-height z-score. AUC – area under the curve.

Annex 7. Stratified Analysis: by Child Sex (Female vs. Male) – Endline

Table S7A. Wasting Prevalences by Child Sex

**Indicator**	**Female** (n=709)	**Male** (n=699)	**p-values**
MUAC	**1.27%** (n=9)<br>(95% CI: 0.45%, 2.09%)	**1.72%** (n=12)<br>(95% CI: 0.75%, 2.68%)	0.489
WHZ	**12.69%** (n=90)<br>(95% CI: 10.24%, 15.14%)	**16.88%** (n=118)<br>(95% CI: 14.10%, 19.66%)	0.027*
Edema	**0.14%** (n=1)<br>(95% CI: 0.0%, 0.41%)	**0.43%** (n=3)<br>(95% CI: 0.0%, 0.9%)	0.310
WHO Definition Wasted: by MUAC **or** WHZ **or** Edema	**13.26%** (n=94)<br>(95% CI: 10.76%, 15.75%)	**17.74%** (n=124)<br>(95% CI: 14.9%, 20.6%)	0.020*
MUACZ	**7.90%** (n=56)<br>(95% CI: 5.91%, 9.88%)	**9.16%** (n=64)<br>(95% CI: 7.02%, 11.29%)	0.398
Wasting by all 4 Measures: (MUAC, WHZ, Edema, MUACZ)	**15.51%** (n=110)<br>(95% CI: 12.85%, 18.18%)	**20.89%** (n=146)<br>(95% CI: 17.87%, 23.90%)	0.009*

CI – confidence interval. MUAC – mid upper arm circumference. MUACZ – mid upper arm circumference z-score. WHZ – weight-for-height z-score.
*p<0.05

Table S7B. ROC Analysis by Child Sex

**ROC Analysis Results**	**Female**	**Male**
Ideal MUAC Threshold	14.4cm	14.4cm
MUAC AUC	0.7353	0.7451
MUAC Sensitivity	78.89%	80.51%
MUAC Specificity	68.17%	68.50%
Ideal MUACZ Threshold	-1	-1
MUACZ AUC	0.7767	0.7457
MUACZ Sensitivity	85.56%	83.05%
MUACZ Specificity	69.79%	66.09%

ROC- receiver operating characteristic. MUAC – mid upper arm circumference. MUACZ – mid upper arm circumference z-score. WHZ – weight-for-height z-score. AUC – area under the curve.

Table S8. Midline Analysis Demographics Table

Sample Characteristics (N=1,482)				
**Demographic Characteristics**	**Total Sample**	Hiran (n=869)	Bay (n=613)	p values
Child Sex, n (%)				
Male	730 (49.26%)	448 (51.55%)	282 (46.00%)	0.035
Female	752 (50.74%)	421 (48.45%)	331 (54.00%)	
Child Age, mean (SD)	35.78 (13.37)	37.49 (13.59)	33.35 (12.66)	<0.001
9-23 months (n, %)	303 (20.45%)	162 (18.64%)	141 (23.00%)	0.040
23-59 months (n, %)	1,179 (79.55%)	707 (81.36%)	472 (77.00%)	
Child Age - 4 categories*				
9-23 months (n, %)	303 (20.45%)	162 (18.64%)	141 (23.00%)	<0.001
24-35 months (n, %)	431 (29.08%)	215 (24.74%)	216 (35.24%)	
36-47 months (n, %)	374 (25.24%)	230 (26.47%)	144 (23.49%)	
48-59 months (n, %)	374 (25.24%)	262 (30.15%)	112 (18.27%)	
**Anthropometric Data,** mean (SD)				
Child MUAC (cm)	14.62 (1.06)	14.48 (0.99)	14.85 (1.12)	<0.001
Weight (kg)	12.44 (2.44)	12.77 (2.47)	11.96 (2.32)	<0.001
Height (cm)	91.30 (10.90)	93.86 (10.75)	87.66 (10.03)	<0.001
**Z Scores**, mean (SD)				
WHZ	-0.74 (1.11)	-1.00 (1.09)	-0.36 (1.04)	<0.001
HAZ	-0.89 (1.43)	-0.49 (1.29)	-1.44 (1.44)	<0.001
MUACZ	-0.81 (0.88)	-0.99 (0.83)	-0.55 (0.88)	<0.001
**Stunting Prevalence,** n (%)				
Not Stunted	1,185 (79.96%)	773 (88.95%)	412 (67.21%)	<0.001
Stunted	297 (20.04%)	96 (11.05%)	201 (32.79%)	
**Wasting Prevalences by Indicator,** n (%) {95% CI}				
MUAC	18 (1.21%) {7.66-19.20%}	6 (0.98%) {0.20%, 1.76%}	12 (1.38%) {0.61%, 2.16%}	0.486
WHZ	189 (12.75%) {11.11-14.55%}	32 (5.22%) {3.46- 6.98%}	157 (18.01%) {15.51%, 20.62%}	<0.001*
Edema	14 (0.94%) {0.56-1.59%}	12 (1.38%) {0.79-2.42%}	2 (0.33%) {0.08-1.30%}	0.039
2013 WHO Wasting Guidelines: by MUAC or WHZ or Edema	209 (14.10%) {12.42-15.97%}	36 (5.87%) {4.01-7.73%}	173 (19.91%) {17.25-22.56%}	<0.001*
MUACZ	123 (8.30%)	28 (4.57%) { 2.91-6.22%}	95 (10.93%) {8.86-13.01%}	<0.001*
Wasting by all 4 Measures: (MUAC, WHZ, Edema, or MUACZ)	{7.00-9.82%}	51 (8.32%) {6.13-10.51%}	203 (23.36%) {20.55-26.17%}	<0.001*

CI – confidence interval. MUAC – mid upper arm circumference. MUACZ – mid upper arm circumference z-score. SD – standard deviation. WHZ – weight-for-height z-score.

Annex S9. Midline Analysis Child Characteristics and Distributions

Figure S9A. Distributions of Child Anthropometric Characteristics: Child Age, Height, MUAC, Weight

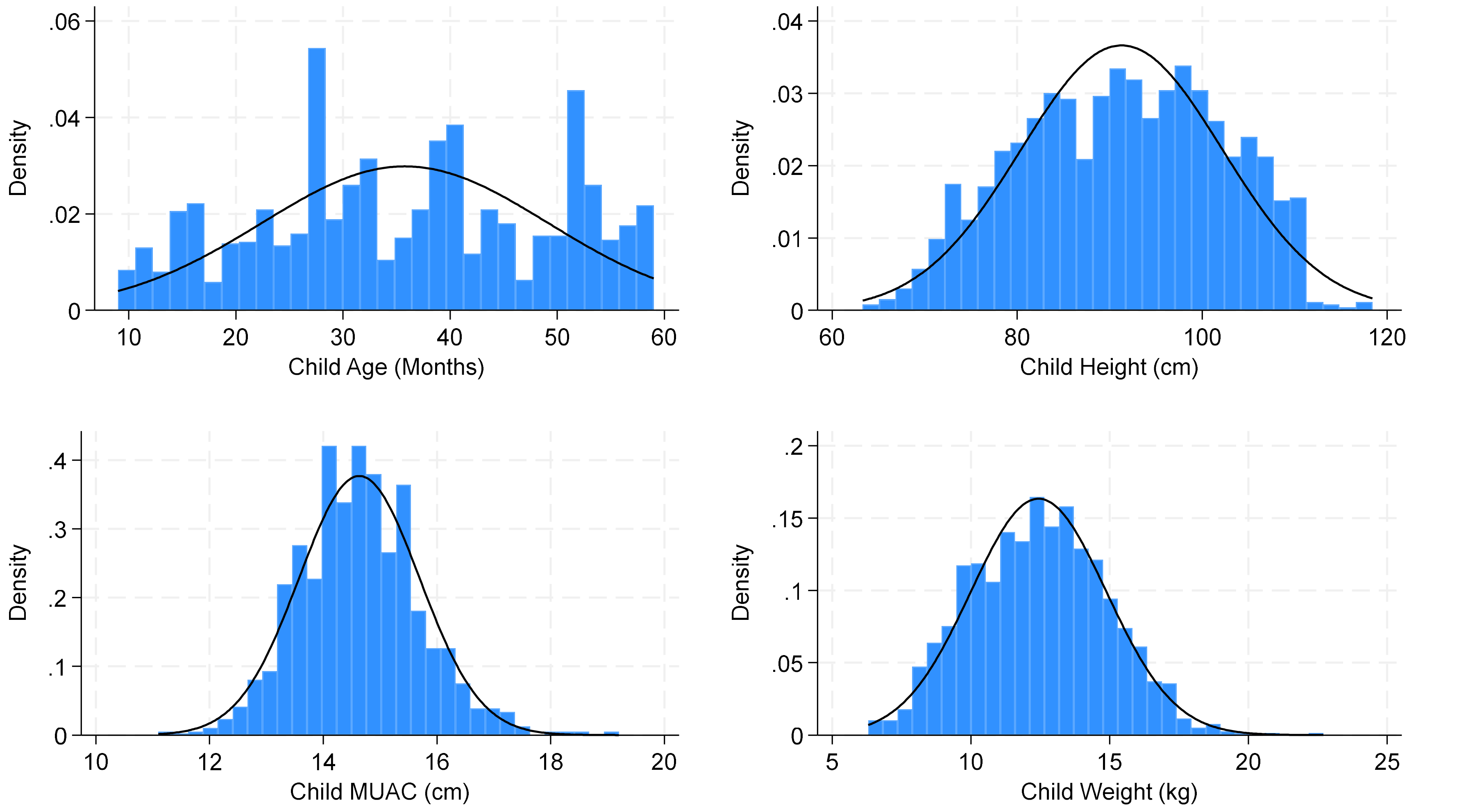

MUAC – mid upper arm circumference. WHZ – weight-for-height z-score.

Figure S9B. Distributions of Child Anthropometric Characteristics: Z-Scores

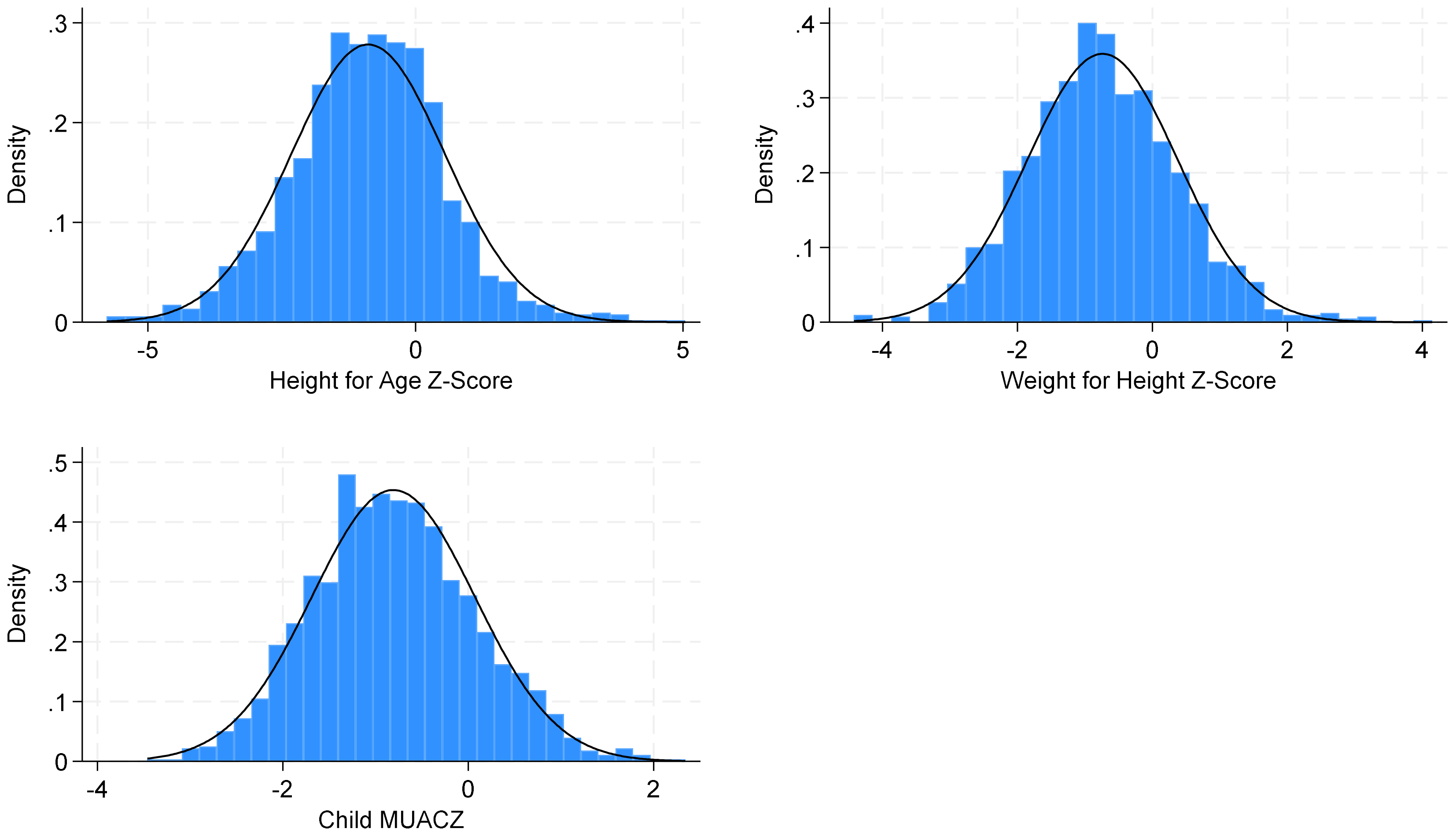

MUACZ – mid upper arm circumference z-score. WHZ – weight-for-height z-score.

Table S9C. Anthropometric Differences by Child Sex

Anthropometric Measurement	Male (n=730)	Female (n=752)	p-value
Wasting Prevalence by MUAC	0.55% (n=4)	1.86% (n=14)	0.021*
Mean MUAC (cm)	14.61cm	14.66cm	0.361
Wasting Prevalence by WHZ	14.93% (n=109)	10.64% (n=80)	0.013*
Mean WHZ	-0.83	-0.65	0.001*

MUAC – mid upper arm circumference. WHZ – weight-for-height z-score.
*p<0.05.

Table S9D. Anthropometric Differences by Child Age (9-23 months vs. 24-59 months)

Anthropometric Measurement	9-23 months (n=303)	24-59 months (n=1,179)	p-value
Wasting Prevalence by MUAC	4.29% (n=13)	0.42% (n=5)	<0.001*
Mean MUAC (cm)	14.07cm	14.78cm	<0.001*
Wasting Prevalence by WHZ	10.56% (n=32)	13.32% (n=157)	0.200
Mean WHZ	-0.49	-0.80	<0.001*

MUAC – mid upper arm circumference. WHZ – weight-for-height z-score.
*p<0.05.

Annex S10. Midline Analysis Pearson's Correlation Figures

Figure S10A. Pearson's Correlation between MUAC and WHZ: ρ=0.5617

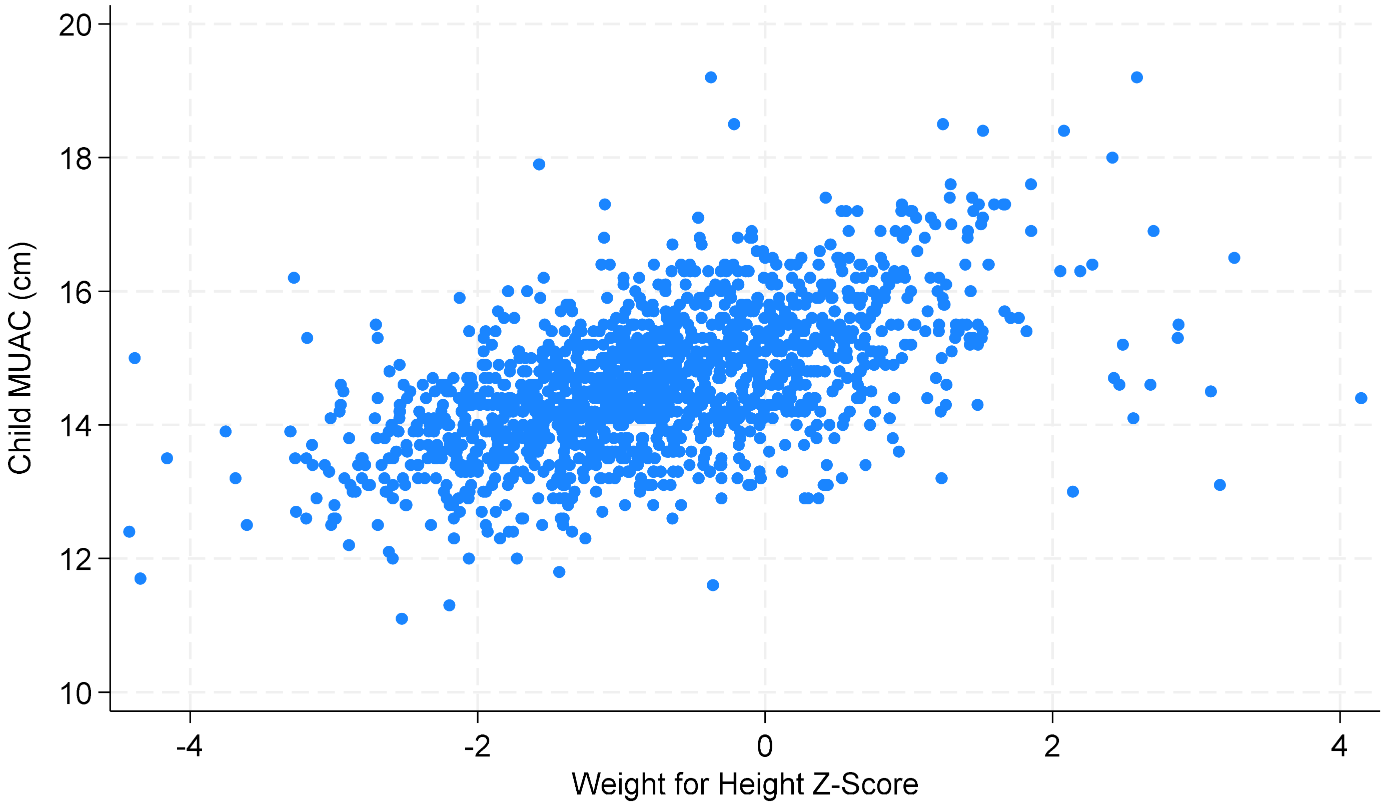

MUAC – mid upper arm circumference. WHZ – weight-for-height z-score.

Figure S10B. Pearson's Correlation between MUAC and MUACZ: ρ=0.8458

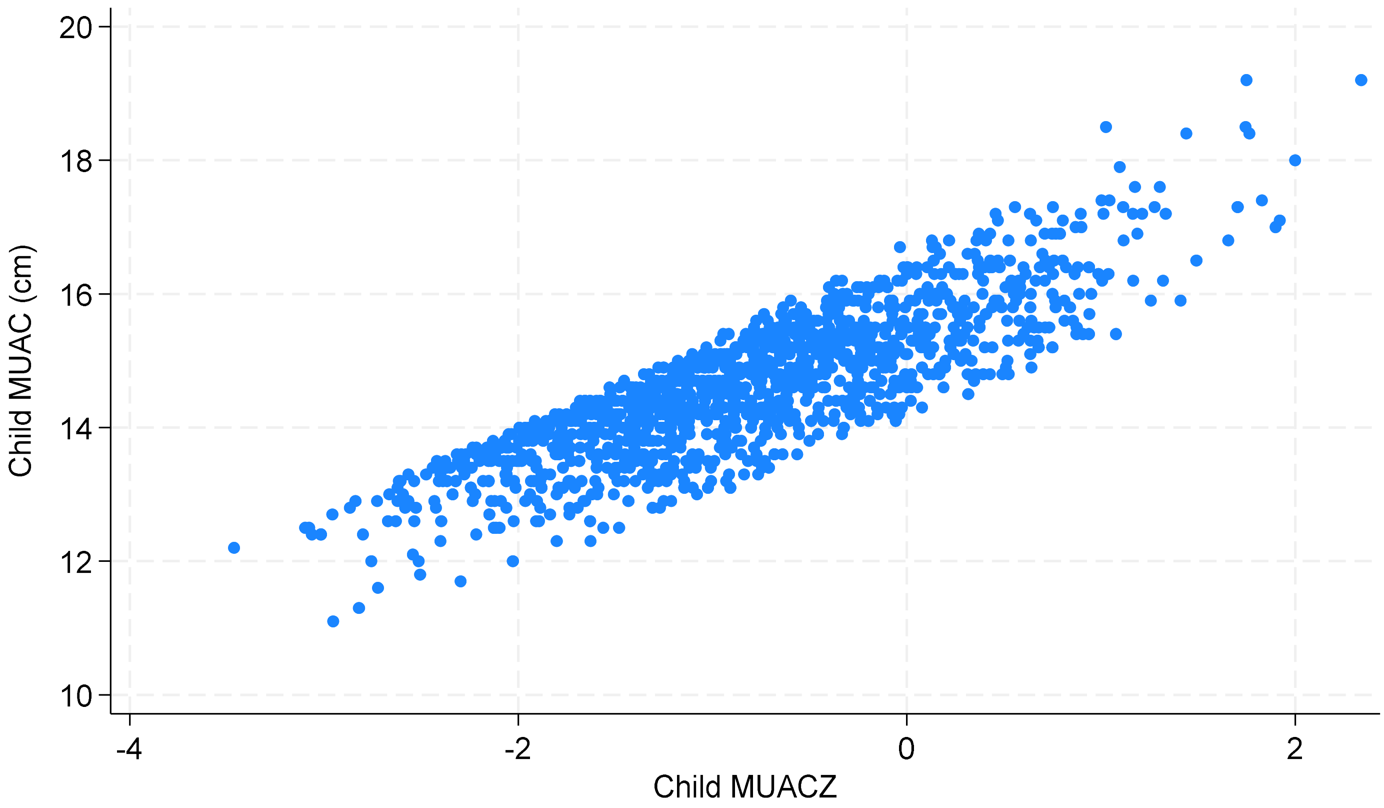

MUAC – mid upper arm circumference. MUACZ – mid upper arm circumference z-score.

Figure S10C. Pearson's Correlation between MUACZ and WHZ: ρ=0.6898

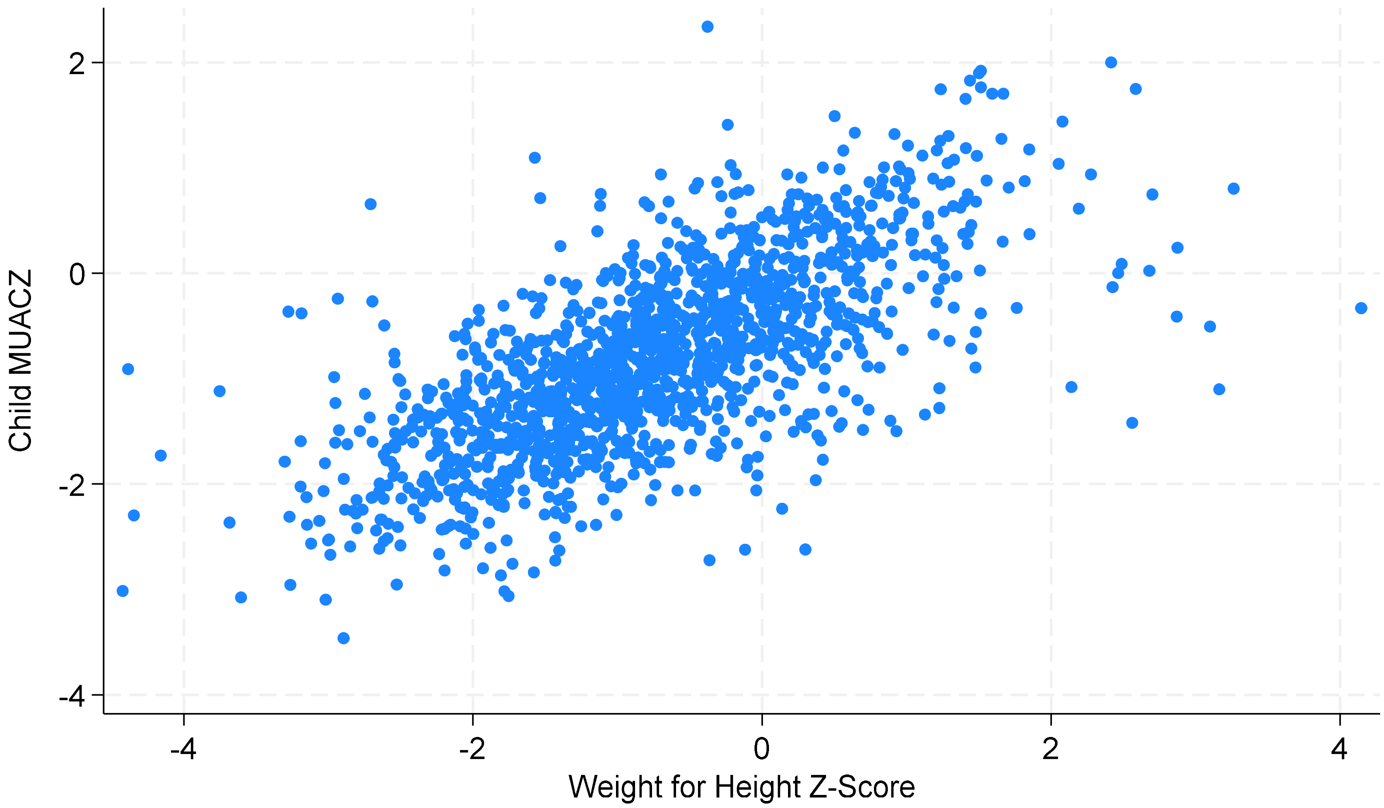

MUACZ – mid upper arm circumference z-score. WHZ – weight-for-height z-score.

Annex S11. Midline Analysis Linear Regression Modelling Results

Table S11A. Regression Modelling for Endline Sample

	**Model 1** β (95% CI)	**Model 2** β (95% CI)	**Model 3** β (95% CI)	**Model 4** β (95% CI)	**Model 5** β (95% CI)	**Model 6** β (95% CI)	**Model 7** β (95% CI)	**Model 8** β (95% CI)
MUAC	0.59‡ (0.55, 0.63)	0.70‡ (0.66, 0.74)	0.72‡ (0.68, 0.76)	0.72‡ (0.68, 0.76)	0.70‡ (0.66, 0.74)	-	-	0.71‡ (0.67, 0.75)
Age (continuous)	-	(-0.033)‡ (-0.037, -0.030)	-0.032‡ (-0.035,-0.029)	-0.030‡ (-0.033, -0.026)	-0.028‡ (-0.032, -0.025)	-	-0.001 (-0.004, 0.002)	-
Sex (Female)	-	-	0.19‡ (0.11, 0.27)	0.33‡ (0.18, 0.48)	0.32‡ (0.17, 0.47)	-	0.072 (-0.008, 0.152)	0.18‡ (0.10, 0.26)
Stunting (Stunted vs. not stunted)	-	-	0.48‡ (0.37, 0.58)	0.48‡ (0.38, 0.58)	0.43‡ (0.32, 0.54)	-	0.44‡ (0.34, 0.55)	0.49‡ (0.38, 0.59)
Age * Sex	-	-	-	-0.18* (-0.34, -0.01)	-0.18* (-0.35, -0.02)	-	-	-
Region (Bay vs Hiran)	-	-	-	-	0.15‡ (0.064, 0.24)		0.16‡ (0.07, 0.24)	-
MUACZ	-	-	-	-	-	0.87‡ (0.82, 0.92)	0.85‡ (0.80, 0.90)	-
Age (reference category 9-23 months)	-	-	-	-	-	-	-	-
24-35 months	-	-	-	-	-	-	-	-0.45 (-0.57, -0.33)
36-47 months	-	-	-	-	-	-	-	-0.79 (-0.91, -0.66)
48-59 months	-	-	-	-	-	-	-	-1.14 (-1.27, -1.71)
**Model R²**	**0.3156**	**0.4664**	**0.5009**	**0.5025**	**0.5063**	**0.4758**	**0.5133**	**0.4899**

CI – confidence interval. MUAC – mid upper arm circumference. MUACZ – mid upper arm circumference z-score. WHZ – weight-for-height z-score.

*p<0.05. †p<0.01. ‡p<0.001.

Note: MUAC and MUACZ were modelled as continuous variables. Sex, stunting, and region were modelled as dichotomous variables. Age was modelled as both a continuous variable and a categorical variable, depending on the model.

Note on Model 8: Linear combinations for this regression model indicated that all categories were statistically significant from each other and all p<0.001.

Annex S12. Midline Analysis Concordance Results

Table S12A. Entire Sample (N=1,482) Concordance Pairs: Wasted vs. Not Wasted

Pair of Wasting Indicators	Kappa	Strength of Concordance
MUAC & WHZ	0.0662	None-slight
MUAC & MUACZ	0.2102	Fair
MUACZ & WHZ	0.3871	Fair

MUAC – mid upper arm circumference. MUACZ – mid upper arm circumference z-score. WHZ – weight-for-height z-score.

Table S12B. Entire Sample (N=1,482) Concordance Pairs: Normal vs. MAM vs. SAM

Pair of Wasting Indicators	Kappa	Strength of Concordance
MUAC & WHZ	0.0487	None-slight
MUAC & MUACZ	0.1681	Fair
MUACZ & WHZ	0.3499	Fair

MAM – moderate acute malnutrition. MUAC – mid upper arm circumference. MUACZ – mid upper arm circumference z-score. WHZ – weight-for-height z-score. SAM – severe acute malnutrition.

Table S12C. Concordance Pairs Stratified by Region: Wasted vs. Not Wasted

Pair of Wasting Indicators	Bay Kappa	Hiran Kappa	Total
MUAC & WHZ	0.1973	0.0344	0.0662
MUAC & MUACZ	0.2228	0.2048	0.2102
MUACZ & WHZ	0.3693	0.3752	0.3871

MUAC – mid upper arm circumference. MUACZ – mid upper arm circumference z-score. WHZ – weight-for-height z-score.

Table S12D. Concordance Pairs Stratified by Age (9-23 months vs 24-59 months): Wasted vs. Not Wasted

Pair of Wasting Indicators	9-23 months Kappa	24-59 months Kappa	Total
MUAC & WHZ	0.2663	0.0166	0.0662
MUAC & MUACZ	0.7197	0.0827	0.2102
MUACZ & WHZ	0.3614	0.3909	0.3871

MUAC – mid upper arm circumference. MUACZ – mid upper arm circumference z-score. WHZ – weight-for-height z-score.

Table S12E. Number of Children Diagnosed as Wasted Using MUAC and/or WHZ Criteria

		**Child Wasting by WHZ**		
		Not wasted	Wasted	**Total**
**Child Wasting by MUAC**	Not wasted	1284	180	1464
	Wasted	9	9	18
	**Total**	1293	189	1482

MUAC – mid upper arm circumference. MUACZ – mid upper arm circumference z-score. WHZ – weight-for-height z-score.

Table S12F. Number of Children Diagnosed as Normal vs. MAM vs. SAM Using MUAC and/or WHZ Criteria

		**Child Wasting by WHZ**			
		SAM	MAM	Normal	**Total**
**Child Wasting by MUAC**	SAM	0	2	0	2
	MAM	2	5	9	16
	Normal	20	160	1284	1464
	**Total**	22	167	1293	1482

MAM – moderate acute malnutrition. MUAC – mid upper arm circumference. MUACZ – mid upper arm circumference z-score. WHZ – weight-for-height z-score. SAM – severe acute malnutrition.

Table S12G. Number of Children Diagnosed as Wasted Using MUAC and/or MUACZ Criteria

		Child Wasting by MUACZ		
		Not wasted	Wasted	**Total**
**Child Wasting by MUAC**	Not wasted	1357	107	1464
	Wasted	2	16	18
	**Total**	1359	123	1482

MUAC – mid upper arm circumference. MUACZ – mid upper arm circumference z-score. WHZ – weight-for-height z-score.

Table S12H. Number of Children Diagnosed as Normal vs. MAM vs. SAM Using MUAC and/or MUACZ Criteria

		Child Wasting by MUACZ			
		SAM	MAM	Normal	**Total**
**Child Wasting by MUAC**	SAM	0	2	0	2
	MAM	4	10	2	16
	Normal	2	105	1357	1464
	**Total**	6	117	1359	1482

MAM – moderate acute malnutrition. MUAC – mid upper arm circumference. MUACZ – mid upper arm circumference z-score. WHZ – weight-for-height z-score. SAM – severe acute malnutrition.

Table S12I. Number of Children Diagnosed as Wasted Using MUACZ and/or WHZ Criteria

		Child Wasting by WHZ		
		Not wasted	Wasted	**Total**
**Child Wasting by MUACZ**	Not wasted	1240	119	1359
	Wasted	53	70	123
	**Total**	1293	189	1482

MUAC – mid upper arm circumference. MUACZ – mid upper arm circumference z-score. WHZ – weight-for-height z-score.

Table S12J. Number of Children Diagnosed as Normal vs. MAM vs. SAM Using MUACZ and/or WHZ Criteria

		Child Wasting by WHZ			
		SAM	MAM	Normal	**Total**
**Child Wasting by MUACZ**	SAM	3	1	2	6
	MAM	11	55	51	117
	Normal	8	111	1240	1359
	**Total**	22	167	1293	1482

MAM – moderate acute malnutrition. MUAC – mid upper arm circumference. MUACZ – mid upper arm circumference z-score. WHZ – weight-for-height z-score. SAM – severe acute malnutrition.

Annex S13. Midline Full Sample ROC Analysis

Table S13A. Current MUAC Thresholds, Sensitivity, Specificity, AUC – Midline

**Full Sample (N=1,482)**			
**Current Threshold**	**Sensitivity**	**Specificity**	**AUC**
MUAC < 12.5cm	4.76%	99.30%	0.520
MUACZ < -2	37.04%	95.90%	0.665

MUAC – mid upper arm circumference. MUACZ – mid upper arm circumference z-score. WHZ – weight-for-height z-score. AUC – area under the curve.

Table S13B. Ideal MUAC Thresholds, Sensitivity, Specificity, AUC – Midline

**Age Category**	**Ideal MUAC Threshold**	**Sensitivity**	**Specificity**	**AUC**
Total Sample	14.3cm	76.19%	69.06%	0.726
9-23 months (n=303)	13.7cm	93.75%	67.90%	0.808
24-35 months (n=431)	14.2cm	79.49%	73.98%	0.767
36-47 months (n=374)	14.6cm	88.00%	65.12%	0.766
48-59 months (n=374)	14.7cm	92.65%	65.36%	0.790

MUAC – mid upper arm circumference. MUACZ – mid upper arm circumference z-score. WHZ – weight-for-height z-score. AUC – area under the curve.

Table S13C. Ideal MUACZ Thresholds, Sensitivity, Specificity, AUC - Midline

**Age Category**	**Ideal MUACZ Threshold**	**Sensitivity**	**Specificity**	**AUC**
Total Sample	-1.1	88.89%	68.21%	0.786
9-23 months (n=303)	-1	93.75%	74.91%	0.843
24-35 months (n=431)	-1.1	82.05%	73.98%	0.780
36-47 months (n=374)	-1.2	82.00%	70.37%	0.762
48-59 months (n=374)	-1.3	89.71%	65.69%	0.777

MUAC – mid upper arm circumference. MUACZ – mid upper arm circumference z-score. WHZ – weight-for-height z-score. AUC – area under the curve.

Annex S14. Stratified Analysis: by Region - Midline

Table S14A. Wasting Prevalence by Region

**Indicator**	**Bay** (n=613)	**Hiran** (n=869)	**p-values**
MUAC	**0.98%** (n=6)<br>(95% CI: 0.20%, 1.76%)	**1.38%** (n=12)<br>(95% CI: 0.61%, 2.16%)	0.486
WHZ	**5.22%** (n=32)<br>(95% CI: 3.46%, 6.98%)	**18.01%** (n=157)<br>(95% CI: 15.51%, 20.62%)	<0.001*
Edema	**1.38%** (n=12)<br>(95% CI: 0.79%,2.42%)	**0.33%** (n=2)<br>(95% CI: 0.08%, 1.30%}	0.039
WHO Definition Wasted:<br>by MUAC or WHZ or Edema	**5.87%** (n=36)<br>(95% CI: 4.01%, 7.73%)	**19.91%** (n=173)<br>(95% CI: 17.25%, 22.56%)	<0.001*
MUACZ	**4.57%** (n=28)<br>(95% CI: 2.91%, 6.22%)	**10.93%** (n=95)<br>(95% CI: 8.86%, 13.01%)	<0.001*
Wasting by all 4 Measures:<br>by MUAC, WHZ, Edema, or MUACZ	**8.32%** (n=51)<br>(95% CI: 6.13%, 10.51%)	**23.36%** (n=203)<br>(95% CI: 20.55%, 26.17%)	<0.001*

CI – confidence interval. MUAC – mid upper arm circumference. MUACZ – mid upper arm circumference z-score. WHZ – weight-for-height z-score. AUC – area under the curve. *p<0.05.

Table S14B. ROC Analysis by Region

**ROC Analysis Results**	**Bay**	**Hiran**
Ideal MUAC Threshold	14.4cm	14.3cm
MUAC AUC	0.7644	0.7108
MUAC Sensitivity	84.38%	75.16%
MUAC Specificity	68.50%	66.99%
Ideal MUACZ Threshold	-0.9	-1.2
MUACZ AUC	0.7939	0.7589
MUACZ Sensitivity	90.62%	84.08%
MUACZ Specificity	68.16%	67.70%

AUC – area under the curve. CI – confidence interval. MUAC – mid upper arm circumference. MUACZ – mid upper arm circumference z-score. WHZ – weight-for-height z-score. ROC – receiver operating characteristic.

Annex S15. Stratified Analysis: by Child Age (9-23 mo. vs 24-59 mo.) - Midline

Table S15A. Wasting Prevalences by Child Age (9-23 months vs 24-59 months)

**Indicator**	**9-23 months** (n=303)	**24-59 months** (n=1,179)	**p-values**
MUAC	**4.29%** (n=13)<br>(95% CI: 2.01%, 6.57%)	**0.42%** (n=5)<br>(95% CI: 0.05%, 0.80%)	<0.001*
WHZ	**10.56%** (n=32)<br>(95% CI: 7.10%, 14.02%)	**13.32%** (n=157)<br>(95% CI: 11.38%, 15.26%)	0.200
Edema	**0.33%** (n=1)<br>(95% CI: 0.05%, 2.32%)	**1.10%** (n=13)<br>(95% CI: 0.64%, 1.89%)	0.215
WHO Definition Wasted:<br>by MUAC or WHZ or Edema	**12.54%** (n=38)<br>(95% CI: 8.81%, 16.27%)	**14.50%** (n=171)<br>(95% CI: 12.49%, 16.51%)	0.381
MUACZ	**5.61%** (n=17)<br>(95% CI: 3.02%, 8.20%)	**8.99%** (n=106)<br>(95% CI: 7.36%, 10.62%)	0.057
Wasting by all 4 Measures:<br>by MUAC, WHZ, Edema, or MUACZ	**13.20%** (n=40)<br>(95% CI: 9.39%, 17.01%)	**18.15%** (n=214)<br>(95% CI: 15.95%, 20.35%)	0.041*

CI – confidence interval. MUAC – mid upper arm circumference. MUACZ – mid upper arm circumference z-score. WHZ – weight-for-height z-score. AUC – area under the curve. *p<0.05.

Table S15B. ROC Analysis by Child Age (9-23 months vs 24-59 months)

**ROC Analysis Results**	**9-23 months**	**24-59 months**
Ideal MUAC Threshold	13.7cm	14.5cm
MUAC AUC	0.8082	0.7428
MUAC Sensitivity	93.75%	82.80%
MUAC Specificity	67.90%	65.75%
Ideal MUACZ Threshold	-1 SD	-1.1 SD
MUACZ AUC	0.8433	0.7741
MUACZ Sensitivity	93.75%	89.71%
MUACZ Specificity	74.91%	65.66%

AUC – area under the curve. CI – confidence interval. MUAC – mid upper arm circumference. MUACZ – mid upper arm circumference z-score. WHZ – weight-for-height z-score. ROC – receiver operating characteristic.

Annex S16. Stratified Analysis: by Child Sex (Female vs. Male) – Endline

Table S16A. Wasting Prevalences by Child Sex

**Indicator**	**Female** (n=752)	**Male** (n=730)	**p-values**
MUAC	**1.86%** (n=14)<br>(95% CI: 0.89%, 2.83%)	**0.55%** (n=4)<br>(95% CI: 0.01%, 1.08%)	0.021*
WHZ	**10.64%** (n=80)<br>(95% CI: 8.43%, 12.84%)	**14.93%** (n=109)<br>(95% CI: 12.35%, 17.52%)	0.013*
Edema	**1.20%** (n=9)<br>(95% CI: 0.42%, 1.97%)	**0.68%** (n=5)<br>(95% CI: 0.08%, 1.28%)	0.308
WHO Definition Wasted: by MUAC **or** WHZ **or** Edema	**12.50%** (n=94)<br>(95% CI: 10.14%, 14.86%)	**15.75%** (n=115)<br>(95% CI: 13.11%, 18.40%)	0.072
MUACZ	**7.85%** (n=59)<br>(95% CI: 5.92%, 9.77%)	**8.77%** (n=64)<br>(95% CI: 6.72%, 10.82%)	0.520
Wasting by all 4 Measures: (MUAC, WHZ, Edema, MUACZ)	**15.03%** (n=113)<br>(95% CI: 12.47%, 17.58%)	**19.32%** (n=141)<br>(95% CI: 16.45%, 22.18%)	0.029*

CI – confidence interval. MUAC – mid upper arm circumference. MUACZ – mid upper arm circumference z-score. WHZ – weight-for-height z-score. AUC – area under the curve.
*p<0.05

Table S16B. ROC Analysis by Child Sex

**ROC Analysis Results**	**Female**	**Male**
Ideal MUAC Threshold	14.2cm	14.3cm
MUAC AUC	0.7253	0.7383
MUAC Sensitivity	71.25%	78.90%
MUAC Specificity	73.81%	68.76%
Ideal MUACZ Threshold	-1	-1.1
MUACZ AUC	0.7811	0.7872
MUACZ Sensitivity	90.00%	91.74%
MUACZ Specificity	66.22%	65.70%

AUC – area under the curve. CI – confidence interval. MUAC – mid upper arm circumference. MUACZ – mid upper arm circumference z-score. WHZ – weight-for-height z-score. ROC – receiver operating characteristic.

Annex S17. Description of Bay and Hiran Regions of Somalia

The Integrated Food Security Phase Classification (IPC) estimates that 1.25 million people are living in Bay, 37% of whom are living in crisis to emergency phases of food insecurity. The estimated population of Hiran is 500,000, 20% of whom are living in crisis to emergency phases of food insecurity. According to the United Nations Refugee Agency, in 2023, there were 555,000 new displacements from Hiran primarily due to flooding, and 276,000 new displacements from Bay due to both drought and flooding. These two regions are also receiving a large proportion of displaced persons from other regions in Somalia.

Annex S18. Detailed Description of CashPlus for Nutrition Study Protocol

The CashPlus for Nutrition Program, funded by the Bureau for Humanitarian Assistance (BHA), is a 6-month humanitarian program in Somalia. Save the Children is implementing this program and Johns Hopkins University is studying the effectiveness and cost-effectiveness of 3 cash interventions for preventing malnutrition (wasting) among children under 5 years of age (CU5) and their mothers. The study was conducted from May 2023 to January 2024 in Bay and Hiran, two regions of Somalia with high wasting prevalence. Villages were selected for the study based on size and location and participants were cluster-randomized at the village level. Eligible participants were mothers of CU5 who were enrolled in the BHA program, were not wasted by mid upper arm circumference (MUAC) in the last year, and had no wasting by MUAC at baseline data collection in May-June 2023. Cash amounts for study arms were determined based on the Minimum Expenditure Basket, the amount needed to meet an average household's nutritional needs for an entire month. Households in Bay received a base cash amount of $90 and households in Hiran received $70. Participants received cash monthly for 6 months. Participants were cluster-randomized into the following arms:

- Arm 1: Received Cash Assistance only
  - Bay: $90
  - Hiran: $70
- Arm 2: Received Cash + SBCC (Social Behavior Change Communication, such as consultations for mothers, support groups on health and nutrition topics, etc.)
  - Bay: $90
  - Hiran: $70
- Arm 3: Received Cash + Top-up Cash:
  - Bay: $90 + $35 = $125
  - Hiran: $70 + $35 = $105

Household survey data was collected from study participants at Baseline, Midline (3 months), and Endline (6 months), including questions on demographic information, household assets, food insecurity, child sickness, and diet. A sample of participants from each arm was selected to participated in qualitative focus groups to understand participants' perspectives on program functioning and satisfaction, community attitudes towards health-seeking and wasting treatment, and how participants used their cash. Study results are currently being analyzed and prepared for publication.

Annex S19. Additional Methods: Analysis Approach

The CashPlus for Nutrition study endline data was downloaded from Kobo Toolbox (KoboCollect;v2022.4.4) and the KoboCollect application. Data was imported and cleaned in STATA. Data were recoded into numeric and string variables as needed. Categorical wasting variables were constructed using the WHO-established clinical cutoffs (found in Box 1 in the manuscript). As described in the paper, data were excluded for not meeting age criteria (>59 months) and for biologically implausible values, leading to a final analytical dataset of 1,408 children.

We ran descriptive statistics on all demographic and anthropometric variables of interest, looking at the distribution of region, sex, age, stunting, MUAC, and z-scores in our study population. Graphs of distributions can be found in the Annex: all variables maintained a fairly normal distribution except for child age, with clusters around 28 and 59 months. We calculated mean, standard, deviation, median, and range for all child characteristics. To examine differences in mean characteristics by child sex, region, age categories, and wasting prevalence, we used paired t-tests. To examine differences in wasting prevalence by child characteristics like sex, age categories, and region, we used chi-squared tests. Wasting prevalences for each anthropometric indicator were calculated using chi-squared tests to generate percentages, 95% confidence intervals and the associated p-values.

Regression modelling was conducted using WHZ as a continuous outcome to examine its relationship with continuous MUAC and other variables of interest (age, sex, stunting, and region). Regression models met assumptions for linearity, independence, and normality; however, regression models were employed with robust variance. All reported models in are found in Table S3A.

To evaluate concordance between anthropometric indicators, we treated WHZ as an evaluative reference standard, since this criterion diagnoses the largest proportion of children as wasted. Tabulations of agreement between pairs of WHZ wasting, MUAC wasting, and MUACZ wasting, and their associated kappa values were reported.

Receiver operating characteristic (ROC) analyses treated WHZ as the “reference standard” and evaluated increasing thresholds of MUAC increasing by 0.1cm and MUACZ increasing by 0.1 units. These increments for MUAC were chosen to reflect the precision with which MUAC was measured in the field by study enumerators (to the nearest 0.1cm). MUACZ increments of 0.1 were chosen for ease of categorization. When evaluating what thresholds of MUAC and MUACZ were “ideal”, we first prioritized maximizing the area under the curve (AUC). When two thresholds produced similar AUC values, we prioritized maximizing the sensitivity over specificity. Ideally we want to identify and treat as many wasted children as possible and there is no harm in providing supplemental nutrition and wasting treatment for a healthy child.

Stratified analyses were conducted across region, age category (9-23 months vs. 24-59 months), and child sex. Stratified analyses included chi-squared tests to examine differences in wasting prevalence using each indicators (MUAC, MUACZ, WHZ, edema). Stratified analyses also compared ROC analyses for each subgroup, examining differences in “ideal” MUAC or MUACZ values by group.

These methods were replicated for the midline timepoint of the study, with a final analytical dataset of 1,482 children. Midline results are found in Annexes S8-S16.

MUAC – mid upper arm circumference. WHZ – weight-for-height z-score. MUACZ – mid upper arm circumference z-score.

Annex S20. Comparison of Wasting Measurement Indicators

Measure	Advantages	Disadvantages	Implementation Needs/Considerations
Current MUAC Threshold (MUAC <12.5cm)	Currently in practice<br><br>No additional retraining/resources	Heavily underestimates true wasting burden<br><br>Misclassifies older children (24-59 months) at a higher rate	N/A
Age-stratified MUAC threshold at 24 months	Identifies more wasted children than current MUAC thresholds<br><br>Utilizes existing supply of MUAC tape<br><br>Ease of measurement	Minimal retraining for CHWs<br><br>Susceptible to inaccurate age data	Retraining CHWs<br><br>New policy requiring government and NGO support<br><br>Increased demand for wasting treatment infrastructure and supply
MUACZ	Identifies more wasted children than current MUAC thresholds<br><br>Ease of measurement	Lower sensitivity than other alternatives<br><br>Retraining for CHWs<br><br>Identifies additional children who may not be wasted using WHZ or MUAC<br><br>Susceptible to inaccurate age data	MUACZ tape supply<br><br>Retraining CHWs<br><br>New policy requiring government and NGO support<br><br>Increased demand for wasting treatment infrastructure and supply
WHZ	Identifies all wasted children<br><br>Most accurate wasting measurement<br><br>Aligns with current global recommendations	Costly supplies (compared to take)<br><br>Complicated measurements<br><br>Significant retraining for CHWs	Length boards and scale supply<br><br>Retraining CHWs<br><br>New policy requiring government and NGO support<br><br>Increased demand for wasting treatment infrastructure and supply
